# Trichostatin A-Mediated Epigenetic Modulation Predominantly Triggers Transcriptomic Alterations in the Ex Vivo Expanded Equine Chondrocytes

**DOI:** 10.3390/ijms232113168

**Published:** 2022-10-29

**Authors:** Tomasz Ząbek, Wojciech Witarski, Tomasz Szmatoła, Sebastian Sawicki, Justyna Mrozowicz, Marcin Samiec

**Affiliations:** 1 Department of Animal Molecular Biology, National Research Institute of Animal Production, Krakowska 1 Street, 32-083 Balice, Poland; 2 University Centre of Veterinary Medicine, University of Agriculture in Kraków, Mickiewicza 24/28, 30-059 Kraków, Poland; 3 Department of Reproductive Biotechnology and Cryoconservation, National Research Institute of Animal Production, Krakowska 1 Street, 32-083 Balice, Poland

**Keywords:** equine chondrocytes, 5-AZA-dc and TSA epigenetic modifiers, transcriptome

## Abstract

Epigenetic mechanisms of gene regulation are important for the proper differentiation of cells used for therapeutic and regenerative purposes. The primary goal of the present study was to investigate the impacts of 5-aza-2′ deoxycytidine (5-AZA-dc)- and/or trichostatin A (TSA)-mediated approaches applied to epigenomically modulate the ex vivo expanded equine chondrocytes maintained in monolayer culture on the status of chondrogenic cytodifferentiation at the transcriptome level. The results of next-generation sequencing of 3′ mRNA-seq libraries on stimulated and unstimulated chondrocytes of the third passage showed no significant influence of 5-AZA-dc treatment. Chondrocytes stimulated with TSA or with a combination of 5-AZA-dc+TSA revealed significant expressional decline, mainly for genes encoding histone and DNA methyltransferases, but also for other genes, many of which are enriched in canonical pathways that are important for chondrocyte biology. The TSA- or 5-AZA-dc+TSA-induced upregulation of expanded chondrocytes included genes that are involved in histone hyperacetylation and also genes relevant to rheumatoid arthritis and inflammation. Chondrocyte stimulation experiments including a TSA modifier also led to the unexpected expression incrementation of genes encoding HDAC3, SIRT2, and SIRT5 histone deacetylases and the MBD1 CpG-binding domain protein, pointing to another function of the TSA agent besides its epigenetic-like properties. Based on the transcriptomic data, TSA stimulation seems to be undesirable for chondrogenic differentiation of passaged cartilaginous cells in a monolayer culture. Nonetheless, obtained transcriptomic results of TSA-dependent epigenomic modification of the ex vivo expanded equine chondrocytes provide a new source of data important for the potential application of epigenetically altered cells for transplantation purposes in tissue engineering of the equine skeletal system.

## 1. Introduction

Aging is a common cause of immune-related disorders, in which underlying altered transcriptional and regulatory mechanisms contribute to inflammatory impairment. For instance, rheumatoid arthritis (RA) is an example of an immune-related condition that is a common cause of degeneration of the hyaline cartilage of the joints. Articular cartilage is an aneural and avascular tissue which has limited regenerative capacity. Therefore, in cases of joint defects, regenerative therapy is often the last available procedure to maintain joint function and restore the overall fitness of the limbs. One of the most effective regenerative approaches is to obtain chondrocytes from the healthy surfaces of the patient’s joints and use them in chondrocyte implantation in places with cartilage defects [1]. The basis for this is the availability of a large number of chondrocytes that will not be dedifferentiated in the course of in vitro expansion. Particularly, in the monolayer culture, which is often the choice for rapid chondrocyte expansion, rapid loss of the primary chondrogenic type of cells is observed. In order to diminish the chondrocyte dedifferentiation, a range of biological stimulants have been tested, which are able to influence the activity of transcription factors or target proteins or to modulate enzymes (COX-2), cytokines (TNF-α/β), and associated inflammatory cascades (NF-kB) that are important for the maintenance of differentiation of chondrocytes [2,3,4,5]. Moreover, it was found that epigenetic modifications such as DNA methylation of regulatory elements of genes or the histone code are able to determine the chondrocyte’s fate [6]. A range of studies, including the in vivo cartilage stimulation of the joints and stimulation of pluripotent stem cells, implemented different epigenetic cures influencing the global expression pattern. These cures encompassed such treatment modalities as the strategies based on the use of either a non-selective inhibitor of DNA methyltransferases (DNMTs), one of which is designated as 5-aza-2′ deoxycytidine (5-AZA-dc) and triggers global passive DNA demethylation [7], or trichostatin A (TSA), representing a family of non-selective inhibitors of histone deacetylases (HDACs). TSA has been found to be an antifungal antibiotic primarily isolated from a culture broth of *Streptomyces platensis* [8]. Its main ability is the non-selective blockage of the class I and II mammalian HDACs, whose enzymatic activities are aimed at mitigating the incidence of acetylation levels within nucleosomal core-derived histones followed by transcriptional repression of the genes. In other words, by the onset of the mechanism of competitive inhibition of HDAC isoenzymes, TSA interferes with the HDAC-induced removal of acetyl groups from histones, and as a consequence of hyperacetylation of lysine residues within histones, it facilitates the opening of chromatin for transcriptional factors promoting the initiation of gene expression [8]. As a potent member of non-specific HDAC inhibitors (HDACIs), TSA has also been considered to be an effective anti-cancer drug used for a wide variety of anti-oncogenic treatment modalities within the framework of epigenetic oncological therapeutics [9]. Moreover, based on oncological research, TSA has been shown to correctly prompt the differentiation processes of dedifferentiated cells [10]. Studies comprising stimulation of primary chondrocytes with 5-AZA-dc showed that DNA demethylation facilitates the terminal differentiation of chondrocytes into the hypertrophic stage [11], whereas 5-AZA-dc treatment of human dedifferentiated chondrocytes from osteoarthritis (OA) patients revealed no substantial impact, despite the presence of osteogenic and adipogenic differentiation of the treated cells [12]. In turn, TSA has been found to be a promising therapeutic agent during the in vivo experimental treatment of cartilage disorders and cartilage regeneration [13] due to its anti-inflammatory properties [14] and ability to prevent cartilage degeneration [10]. However, in vitro studies implementing TSA treatment of human bone marrow mesenchymal stem cells (hBMMSCs) showed that TSA inhibited chondrogenic differentiation, making TSA probably not useful for cartilage tissue engineering using hBMMSCs [15].

Due to contradictory results affecting the efficiency of 5-AZA-dc and TSA—epigenetic modulators of chondrogenesis—the aim of our study was to describe transcriptional variation upon 5-AZA-dc and TSA stimulation of expanded equine chondrocytes in monolayer cultures. While 5-AZA-dc exerted no effect at the transcriptome level of expanded chondrocytes, TSA stimulation resulted in the expressional decline of genes encoding methyltransferases and genes which are overrepresented in a range of molecular pathways relevant to chondrogenesis. It seems that TSA stimulation of in vitro expanded chondrocytes is less beneficial regarding chondrogenic capacity in comparison to the reported intraarticular administration of TSA, where it showed a protective effect on the cartilage [13]. Although TSA- and/or 5-AZA-dc-assisted epigenetic transformation of the ex vivo expanded horse chondrocytes gives rise to a decrease in the expression of a wide variety of genes responsible for chondrogenic differentiation, efforts to characterize the genomic and epigenomic signatures of equine cartilage-derived cell lines appear to be required for exploring the capabilities of TSA- and/or 5-AZA-dc-transformed, cartilage-derived somatic cell nuclei to be epigenetically reprogrammed in cloned horse embryos. Estimating the backgrounds pinpointed for epigenetic reprogrammability and molecular dedifferentiability of TSA- and/or 5-AZA-dc-modulated chondrocyte cell nuclei for the needs of generating and multiplying genetically identical equine cloned embryos, conceptuses, and progeny might be shown to be a viable solution that can be applied to modern assisted reproductive technologies based on somatic cell nuclear transfer (SCNT) in horses and other mammalian species.

## 2. Results

### 2.1. Efficiency Alignment of NGS Reads

Next-generation sequencing of 3’ mRNA-seq libraries produced from 2,332,147 to 3,699,592 filtered reads per sample with the efficiency of 80.1–82.7% for uniquely mapped reads, using the EquCab 3.0 version of the horse genome (Table 1).

### 2.2. Differentially Expressed Genes (DEGs) Obtained Using 3′ mRNA-Seq

The comparison among investigated groups (Table 1) produced a list of differentially expressed genes (DEGs). The adjusted *p*-value was less than 0.05 for groups I and II (5-AZA-dc versus 5-AZA-dc+TSA) (Table S1), I and IV (5-AZA-dc versus TSA) (Table S2), II and III (5-AZA-dc+TSA versus control group) (Table S3), and III and IV (control group versus TSA) (Table S4). We found a lack of significant DEGs between cells stimulated with 5-AZA-dc and the control group (I vs. III).

**Figure 1:**
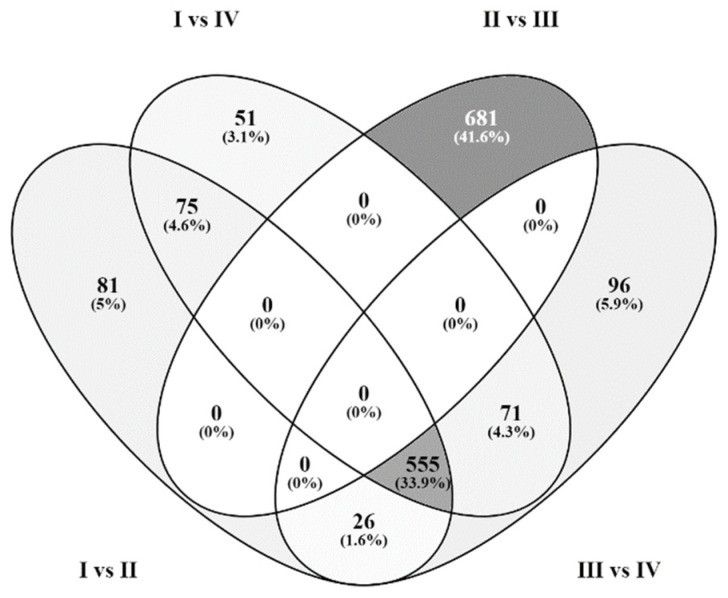
The Venn diagram showing the numbers of common DEGs in four comparisons when upregulated in: group I vs. group IV (5-AZA-dc versus TSA), I vs. II (5-AZA-dc versus 5-AZA-dc+TSA), II vs. III (5-AZA-dc+TSA versus control group) and III vs. IV (control group versus TSA).

**Figure 2:**
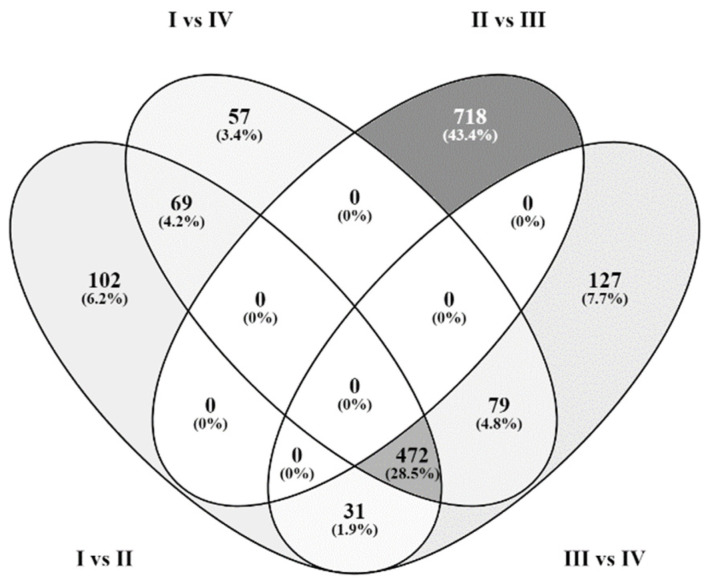
The Venn diagram showing the number of common DEGs in four comparisons when downregulated in: group I vs. group IV (5-AZA-dc versus TSA), I vs. II (5-AZA-dc versus 5-AZA-dc+TSA), II vs. III (5-AZA-dc+TSA versus control group), and III vs. IV (control group versus TSA).

From the list of 1980 DEGs showing a minimum fold change value of at least 1, two major sets of genes were characterized after the stimulation of cells. The first group was represented by 1636 genes upregulated in group I vs. group IV (5-AZA-dc versus TSA), I vs. II (5-AZA-dc versus 5-AZA-dc+TSA), II vs. III (5-AZA-dc+TSA versus control group), and III vs. IV (control group versus TSA) (Figure 1). The second set included 1655 genes which were downregulated in the mentioned comparisons (Figure 2).

The majority of differentially expressed genes between the investigated groups were found in comparisons that included TSA stimulation of cells. Namely, the comparison of DEG lists using the Venny integrative tool (http://bioinfogp.cnb.csic.es/tools/venny/index.html, accessed on 8 August 2022) revealed 681 exclusively upregulated and 718 downregulated genes in group II vs. group III (5-AZA-dc+TSA versus control group) and 555 exclusively upregulated and 472 downregulated genes in I vs. II, I vs. IV, and III vs. IV (5-AZA-dc versus 5-AZA-dc+TSA, 5-AZA-dc versus TSA, and control group versus TSA) (Figure 1 and Figure 2).

### 2.3. General Description of Differentially Expressed Genes upon Applying Chondrocyte Stimulation in Monolayer Culture

**Figure 3:**
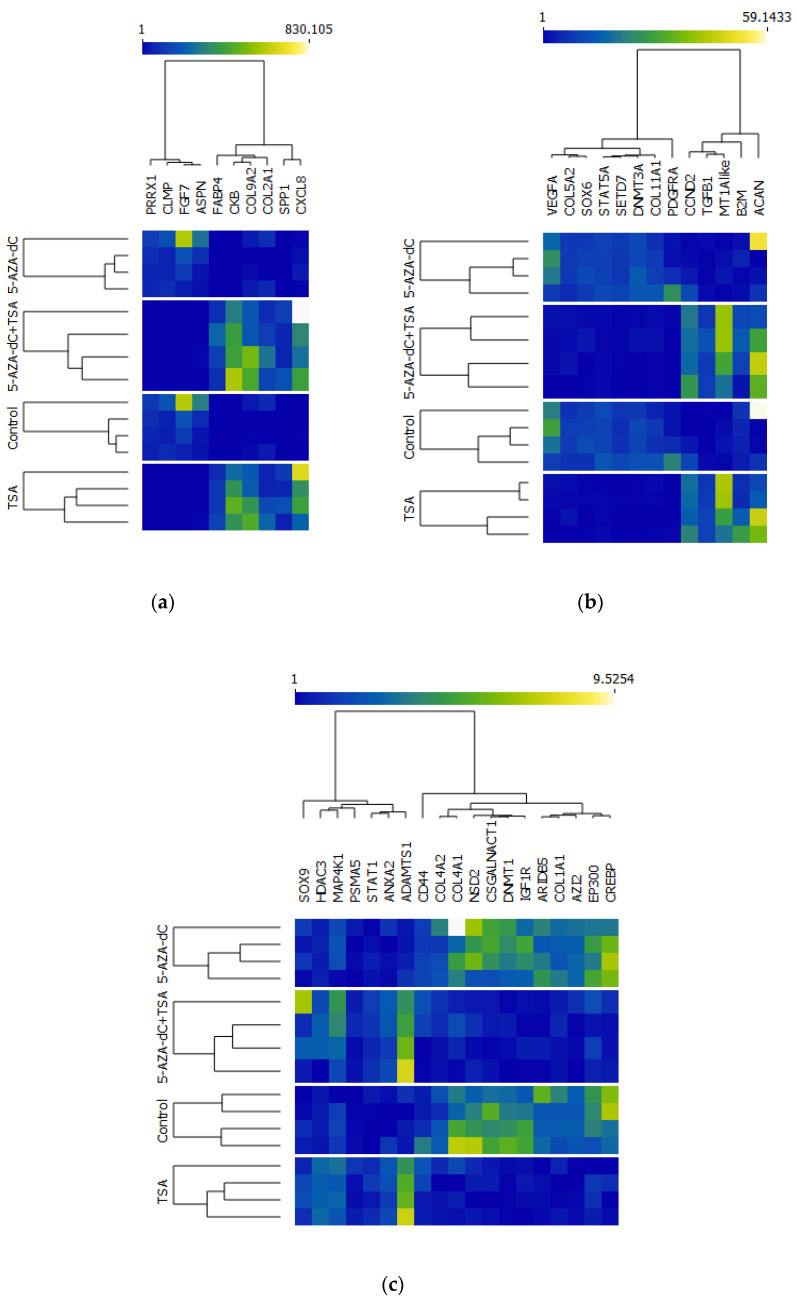
Differential expression results for 42 genes that are important for chondrogenesis and epigenetic modifications, which were validated using quantification with real-time PCR. Heat maps were generated using Orange: Data Mining web available software. Each heat map shows low expression values in blue and high expression values in yellow and white. In each figure, two major clusters are visible: the first represents genes downregulated after TSA or 5-AZA-dc+TSA treatment, and the second includes genes that were upregulated after TSA or 5-AZA-dc+TSA stimulation. (**a**) DEGs with fold changes of expression in the range from 1 to 830.1; (**b**) DEGs with fold changes of expression in the range from 1 to 59.14; (**c**) DEGs with fold changes of expression in the range from 1 to 9.5.

A list of all DEGs with descriptions of their encoding proteins is included in Table S6. Variably expressed genes in this study included loci involved in the functioning of the genetic apparatus during the cell cycle, which are also important for the epigenetic control of transcriptome machinery—e.g., encoding proteins of histone acetyltransferases (HATs), acetylation readers, histone deacetylases (HDACs), histone methyltransferases (HMTs), genes encoding DNA methyltransferases and a methyl-CpG-binding domain [16], and also an equine counterpart of a gene encoding 5-azacytidine-induced protein 2 (AZI2), whose cDNA was first detected in a human cell line after stimulation with the 5-AZA-c demethylation agent [17] (Figure 3). Genes encoding histone methyltransferases (SETD2, SETD7, KMT2E, KMT5A, KMT5B, PRMT7), the BRD1 histone acetylation reader, DNA methyltransferases (DNMT1 and DNMT3A), and AZI2 were downregulated in TSA- or 5-AZA-dc+TSA-stimulated cells in comparison to the control or to chondrocytes stimulated exclusively with 5-AZA-dc. Those encoding CREBBP and EP300 histone acetyltransferases; HDAC3, SIRT2, and SIRT5 histone deacetylases; and methyl-CpG-binding domain protein 1 (MBD1) were upregulated in TSA- or 5-AZA-dc+TSA-stimulated cells (Figure 3). Genes downregulated in TSA- or 5-AZA-dc+TSA-treated cells included also those encoding particular types of chondroproteins (Figure 3) [18]. These were genes encoding collagens (COL1A1, COL3A1, COL4A1, COL5A2, COL8A1, COL11A1, and COL12A1); non-collagenous regulatory (BMP6, MXRA5, TGFB1I1, TGFB3) and structural proteins (ECM2, EFEMP2, FNDC3B); membrane-associated proteins, such as integrins (ITGA1, -5, and -7, and ITGB5), annexin (LOC100052045), chondroitin sulfate N-acetylgalactosaminyltransferase 1 (CSGALNACT1), syndecan (SDC2), and discoidin (DDR2); and one of the proteoglycan proteins—asporin (ASPN) [18]. A set of genes which was also downregulated included those encoding transcriptional factors (TFs) important for chondrogenesis (DLX5, NFIB, NFIX, PRRX1, SOX6, TCF7L1, TRPS1, ZBTB20) [19]. Some of the genes which were upregulated in TSA- or 5-AZA-dc+TSA-stimulated cells versus control cells or cells stimulated with 5-AZA-dc alone included *COL2A1*, *COL9A2*, *ITGA2*, *DCBLD2*, *ANXA2*, *TGFB1*, *ADAMTS1*, *ADAMTS15*, and one gene encoding a chondrogenic transcriptional factor (STAT1) (Figure 3).

### 2.4. Results of Functional Overrepresentation of DEGs Using DAVID Annotation Tools

We have performed the analysis of gene enrichment in GO terms for loci with the fold change thresholds of equal to or above 1 and equal to or below −1. Implementation of a DAVID Functional Annotation Chart (https://david.ncifcrf.gov/tools.jsp, accessed on 4 July 2022) using the horse genome as the background showed significant enrichment of sets of genes involved in pathways in cancer (ecb05200), cell cycle (ecb04110), focal adhesion (ecb04510), ECM–receptor interaction (ecb04512), glycolysis/gluconeogenesis (ecb00010), proteoglycans in cancer (ecb05205), HIF-1 signaling pathway (ecb04066), PI3K-Akt signaling pathway (ecb04151), FoxO signaling pathway (ecb04068), and TGF-beta signaling pathway (ecb04350) (Table S7–S10). A variety of genes contributing to the mentioned pathways were upregulated in the 5-AZA-dc group compared to the 5-AZA-dc+TSA group or TSA group, and these were also upregulated in the control group compared to the TSA group or downregulated in the 5-AZA-dc+TSA group compared to the control group (Tables S7–S10). Moreover, DEGs between particular biological groups in this study were also enriched for the MAPK signaling pathway (ecb04010) (genes were upregulated in cells stimulated with 5-AZA-dc versus cells stimulated with the combination 5-AZA-dc+TSA or with TSA alone; they were also upregulated in controls versus cells stimulated with TSA), fluid shear stress and atherosclerosis (ecb05418) and lysine degradation (ecb00310) (genes were upregulated in cells stimulated with 5-AZA-dc versus with the combination of 5-AZA-dc+TSA, or downregulated in cells stimulated with 5-AZA-dc+TSA versus the control group), and the thyroid hormone signaling pathway (ecb04919) (genes were downregulated in cells stimulated with 5-AZA-dc+TSA versus the control group). Rheumatoid arthritis (ecb05323) and metabolic pathways (ecb01100) were the ones enriched with genes downregulated in the 5-AZA-dc group versus the 5-AZA-dc+TSA or TSA group, and these genes were also downregulated in the control group compared to the TSA group or upregulated in the 5-AZA-dc+TSA group compared to the control group (Tables S7–S10).

### 2.5. Validation in RNA-Seq Results

In order to check for the validity of identified transcript abundance in RNA-seq data, we selected 42 genes which were important for the biological background of the designed experiment (violin plots in Figures S1–S42 in Supplementary Figures). The tendency and significance of differential expression at the mentioned loci were confirmed using quantification via real-time PCR. In general, heat maps representing the transcriptional activity of validated genes showed two major gene clusters relevant to down- or upregulation upon TSA or 5-AZA-dc+TSA treatment of chondrocytes from the third passage (Figure 3).

Contrary to RNA-seq results, real-time PCR showed a lack of significant transcriptional variation in the *CD44* and *PSMA5* loci among all compared groups. A lack of significant differences was also found between 5-AZA-dc+TSA and the control group in *COL4A2* (Figure S15) and EP300 loci (Figure S24) (only the trend); between 5-AZA-dc, TSA, and the control group and TSA in the *STAT1* locus (Figure S38) (only the trend); between 5-AZA-dc and 5-AZA-dc+TSA and between 5-AZA-dc+TSA and the control group in the *HDAC3* locus (Figure S27); and between 5-AZA-dc and 5-AZA-dc+TSA in the *MAP4K1* locus (Figure S29). Moreover, real-time PCR using primers for *DNMT1* revealed significant differences between 5-AZA-dc+TSA and the control group, and the control and the TSA group, at this locus, which were not detected in RNA-seq data. We also checked for transcript abundance of additional markers of chondrogenic differentiation, such as *ACAN* and *SOX9*. Real-time PCR results at the *ACAN* locus revealed nonsignificant downregulation in TSA- or 5-AZA-dc+TSA-stimulated cells, showing only the trend (Figure S1). Relative quantification results of transcriptional variation between groups at the *SOX9* locus were not significant (Figure S37).

## 3. Discussion

5-AZA-dc and TSA are common agents used for studies on the cell cycle and cells’ epigenetic alterations in in vitro systems. In this study, we observed a predominant influence of TSA treatment on transcriptome alterations of chondrocytes in monolayer cultures of the third passage. According to the observed transcriptional variation upon 5-AZA-dc and TSA treatment after the third passage of cultivated cells, the effect of 5-AZA-dc seemed to be marginal in comparison to the TSA treatment. There was a lack of significant differences in transcript abundance between 5-AZA-dc-stimulated cells and the control group. Moreover, *AZI2*, which was found to be a special marker, being upregulated under 5-azacytidine-induced demethylation [17], was underexpressed in TSA- and 5-AZA-dc+TSA-treated cells in our study. The investigated cells of the third passage were less affected by the 5-AZA-dc treatment due to a range of possible factors, such as differences in the proliferative potential between cell lines, and uneven surface coating with the applied matrigel, leading to variability in the phenotype changes due to in vitro handling. It is of note that the blockage of DNA methylation via 5-AZA-dc was reported to be more pronounced in the primary chondrocytes [20]. One possible sign of the 5-AZA-dc activity in the passaged chondrocytes might be observed upregulation of an important osteogenic marker, such as secreted phosphoprotein 1 (*SPP1*), and that of a gene relevant to adipogenic differentiation, e.g., fatty acid binding protein 4 (*FABP4*). It was previously found that human OA chondrocytes pre-cultivated with 5-AZA-dc showed signs of osteogenic and adipogenic differentiation [12]. Nonetheless, the majority of detected transcriptional variation in this study affected genes after the TSA or 5-AZA-dc+TSA treatment of equine chondrocytes. The demethylating effect of TSA seemed to be evident in our study, as genes encoding histone methyltransferases, and those encoding DNA methyltransferases, were downregulated in cells stimulated with TSA or 5-AZA-dc+TSA. However, this effect should be also checked on the DNA methylation level. Moreover, according to the literature, downregulation of DNA methyltransferases by TSA may lead to increased expression of *CREBBP* and *EP300* histone acetyltransferases and *BRD1* (histone acetylation reader), all of which occurred in our study, confirming the effect of TSA histone hyperacetylation reported elsewhere [21]. The exception was that genes encoding *HDAC3*, *SIRT2*, and *SIRT5* histone deacetylases and methyl-CpG-binding domain protein 1 (*MBD1*) belonged to a group of loci that were upregulated in chondrocytes stimulated with TSA or 5-AZA-dc+TSA. We could not find any solid explanation for this opposite effect of TSA, especially for the *HDAC3* and *MBD1* expression increases. Trichostatin A is a common inhibitor of histone deacetylases which possess a zinc-dependent active site, like HDAC3, but SIRT2 and SIRT5 histone deacetylases are in general not affected by TSA [16]. From the biological perspective, the observed expression pattern might be uncoupled from the function of histone deacetylation by *HDAC3*, *SIRT2*, and *SIRT5*, which might also have another biological role, being functionally linked with other genes induced by TSA treatment in expanded chondrocytes. For instance sirtuins themselves have been found to promote MSC chondrogenesis [22] and are important regulators of cartilage homeostasis [23]. In this regard, TSA-related *SIRT2* or *SIRT5* induction could be important for chondrocyte differentiation during expansion. In turn, methyl-CpG-binding domain protein 1 (MBD1) possesses a strong affinity to DNA methylation [24] and should be downregulated under TSA stimulation. The reason for the TSA-related *MBD1* expression increment might have been a diminished demethylation effect of chondrocytes from the third passage, which were nonsignificantly affected by 5-AZA-dc, possibly due to the factors mentioned earlier in the discussion. Therefore, TSA stimulation with an applied dose to expanded chondrocytes would seem to exert heterogeneous effects, rather than being only a demethylation or hyperacetylation agent. However, we did not find any relevant literature about the adverse effects of TSA on the activity of the above-mentioned genes. The aforementioned assumptions need to be further experimentally verified. Regarding the maintenance of the chondrogenic potential of expanded cells, the results we obtained are similar to the ones from work on chondrogenesis of human bone marrow mesenchymal stem cells (hBMMSCs), where TSA treatment resulted in reduced expression of chondrogenesis-related genes [15]. In our study, genes downregulated after TSA or 5-AZA-dc+TSA treatment in expanded chondrocytes were those encoding numerous chondroproteins, such as collagens, non-collagenous regulatory and structural proteins, membrane-associated proteins, and proteoglycans. TSA also downregulated a group of genes encoding transcriptional factors (TFs) that are important for chondrogenesis. Some exceptions included chondral genes such as *COL2A1*, *COL9A2*, *ITGA2*, *DCBLD2*, *ANXA2*, and *TGFB1*, which were upregulated under the influence of TSA or 5-AZA-dc+TSA. *COL2A1* and *COL9A2* genes, of the six mentioned above, were highly expressed under TSA or TSA+5-AZA-dc stimulation. *COL2A1* and *COL9A2* encode components of the extracellular matrix characteristic for mature articular cartilage, which are regulated by the SOX9 master chondrogenic transcription factor in combination with SOX5 and SOX6 (TFs) [25]. In our study, we did not observe significant expression alterations of *SOX9*, despite substantial *COL2A1* and *COL9A2* TSA-related expression incrementation. Moreover, we even detected TSA-related *SOX6* downregulation, pointing to other downstream mechanisms of *COL2A1* and *COL9A2* upregulation under TSA treatment where a range of other TFs could be involved.

It is of note that the transcript abundance of *ACAN*, which is an important marker of chondrocyte differentiation regulated by SOX9 TF [25], was unaffected by TSA treatment and by 5-AZA-dc+TSA treatment of chondrocytes in our study. According to the study on the regulation of type-IX collagen gene expression in human osteoarthritic chondrocytes [26], exposure to potentially and indirectly demethylating agents such as a member of non-selective HDACIs designated as TSA might result in the activation of genes regulated by CpG differential methylation. This, however, needs to be further explored in the target loci using molecular approaches relying on bisulfite-converted DNA. In our study, we also observed TSA-related downregulation of gene groups involved in canonical molecular pathways that are important for the biology of differentiated chondrocytes, such as ECM–receptor interaction [27]; focal adhesion [28]; and HIF-1-, PI3K-Akt-, TGF-beta-, and FOXO- and MAPK signaling pathways [29,30,31,32,33]. Other KEGG pathways with TSA-downregulated genes in our study included glycolysis/gluconeogenesis pathways, which are relevant to glucose uptake during chondrocyte maturation [34]; the fluid shear stress pathway, a relevant one possibly reflecting the role of mechanical loading on cartilage during chondrocyte maturation [35]; and the thyroid hormone signaling pathway, where TSH is involved in terminal differentiation of growth-plate chondrocytes [36]. Some genes which were upregulated under the influence of TSA or 5-AZA-dc+TSA are enriched in rheumatoid arthritis (RA), pointing to an alleged negative outcome of the applied means of stimulation of expanded chondrocytes in the monolayer. These loci include chemokines, matrix metallopeptidase 3 (*MMP3*), and the Fos proto-oncogene AP-1 transcription factor subunit (*FOS*); all of them contribute to molecular pathways involved in RA development [37]. Moreover, TSA- or 5-AZA-dc+TSA-related upregulation of expanded chondrocytes in this report affected other genes involved in pathophysiological remodeling and inflammation, such as those encoding for ADAMTS1 and ADAMTS15 aggrecanases [38], and STAT1, a transcription factor which inhibits chondrocyte maturation in the growth plate [19] and indirectly leads to severe chondrodysplasias [39].

In summary, it must be underlined that two-dimensional (2D) culture under monolayer conditions seems to be the fastest and least expensive method of rapid chondrocyte expansion for the purposes of regenerative and reconstructive medicine. Nevertheless, the ex vivo 2D models used for the proliferation of equine chondrocytes appear to be considered as a potential risk factor that can expedite a loss of phenotypic background specific for cartilage cells (i.e., disappearance of their chondrogenic potential) [40]. For that reason, future investigations are required to estimate the reliability and feasibility of TSA- and/or 5-AZA-dc-dependent approaches to epigenomically modulate the transcriptomic profiles pinpointed for three-dimensional (3D) models of chondrocyte expansion. The latter might provide a more conducive environment for the effective transduction of intracellular signals indispensable for perpetuating the chondrogenic differentiation of cells. Moreover, obtained results in form of transcriptome signatures of ex vivo expanded equine chondrocytes in a 2D model, subjected to TSA- and/or 5-AZA-dc-assisted epigenomic modulation, might contribute to the enhancement in biomedical applicability of epigenetically modified cartilage cells in regenerative medicine. Further, TSA- and/or 5-AZA-dc-modified chondrocyte cell lines might provide a completely new source of epigenetically altered nuclear donor cells (NDCs) usable for future studies on somatic cell cloning of horses. Such SCNT-derived horse clones might be especially suitable for studies on the treatment of a variety of histopathological changes of the skeleton. These can be pre-clinical and clinical studies designed to elaborate the negligibly intrusive procedures of reconstructive medicine and chondroplasty-mediated tissue engineering of the horse skeletal system. Such research could be applied for the treatment of chondrodystrophic and chondrodysplastic lesions evoked either by post-operative intra- and intercartilaginous connective tissue-based adhesions or by accidental and inflicted chondral defects in domestic horses [41,42,43]. At this point, the treatment of both degenerative abnormalities (e.g., chondromalacia patellae) in senescent chondral connective tissue and heritable malformations of the equine chondroskeletal system [44,45] could also be considered. Important pathophysiological transformations which would be another subject in this area include premalignant cartilaginous lesions in specimens afflicted with precancerous chondrocyte hypertrophy or chondrometaplasia (chondromatosis) [46,47,48]. Finally, other interesting ones in this matter are the oncologic disorders that are related to the carcinogenesis of cartilaginous origin such as malignant chondral neoplasms classified as metastatic chondrocyte-mediated cancers and non-malignant chondroskeletal tumors in high-genetic-merit horses displaying tremendous performance rates [49,50,51,52,53].

## 4. Materials and Methods

### 4.1. Short Description of the Research

**Figure 4:**
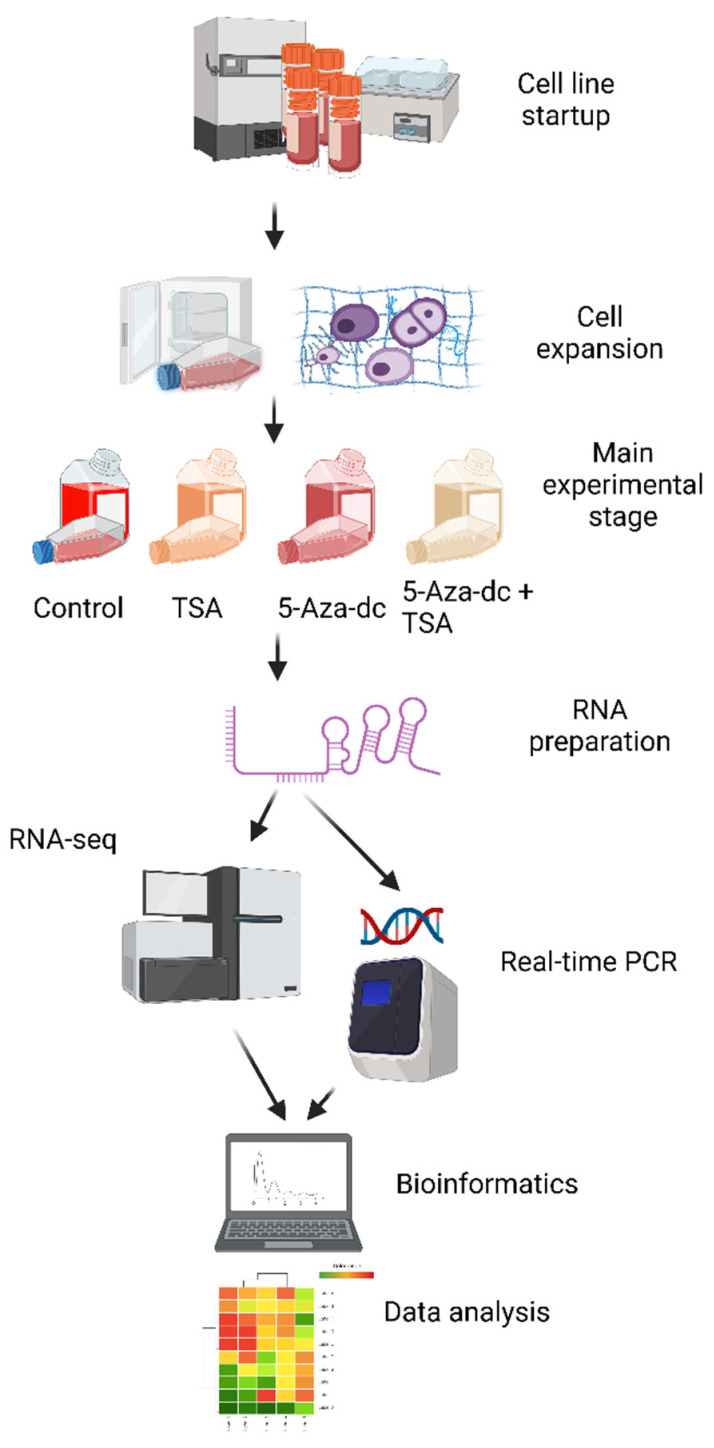
Scheme of the research (created with BioRender.com; license agreement *FU24GOX88E*).

The study included the setup of chondrocytes of the 1st passage and cells’ adaptation to the in vitro conditions. The main stages of the experiments included the epigenetic stimulation of cultivated chondrocytes in the monolayer that reached the 3rd passage, preparation of RNA samples and RNA-seq libraries, NGS sequencing, validation of RNA-seq results, bioinformatics, and data analysis, including genomic annotation and gene overrepresentation tests in gene ontology terms (Figure 4).

### 4.2. Chondrocyte Culture Conditions and Applied Stimulations

The subjects of the study were equine chondrocyte cells preserved at the 1st passage of a monolayer culture stored in DMSO at −80 °C, which were previously developed from chondrocytes obtained post-mortem from the hyaline cartilage shavings of the metacarpal–phalangeal joints (by-product of slaughter material) of four unrelated cold-blood horses aged one and a half years [54]. The refrigeration stage included incubation of cells at 37 °C in DMEM high-glucose medium (Gibco, Grand Island, NY, USA) including 10% fetal bovine serum (FBS), 0.3 mg of GlutaMAX Supplement per 1 mL (Gibco Grand Island, NY, USA), and 1× Primocin (Invivogen, San Diego, CA, USA). Thereafter, chondrocytes were seeded on 10 cm Petri dishes (Eppendorf, Hamburg, Germany) previously coated with Geltrex LDEV-Free Reduced Growth Factor Basement Membrane Matrix (Thermofisher Scientific, Waltham, MA, USA). We introduced coating with Geltrex because matrigel-like compositions were characterized to be some of the most interesting solutions for preventing or delaying the dedifferentiation of cultivated chondrocytes [55]. The complex composition of Geltrex also allows chondrocytes to develop laminin-mediated effects, this being a key component of the extracellular matrix which can promote chondrogenesis [56]. Cells were next considered suitable for further stimulation when able to regenerate from approximately 25% to almost 100% confluency within four days on the same-sized dish. Including all stages of chondrocyte adaptation to the in vitro conditions, the start of the main experiments began with the cultivation of 400,000 cells of the 3rd passage per Petri dish. Chondrocytes of the 3rd passage were subjected to epigenetic stimulation with 5-AZA-dc (Merck, Darmstadt, Germany) (group I), TSA (Merck, Darmstadt, Germany) (group IV), or a combination of both agents (5-AZA-dc+TSA) (group II). The control group included cells exclusively stimulated with DMSO (Merck, Darmstadt, Germany) (group III). The 5-AZA-dc concentration of 25 µg per 1 mL of the culture medium and the TSA concentration of 0.25 µg/mL were used as nontoxic doses in the aforementioned groups. In every experimental setting, equal amounts of DMSO were used as a solute carrier and as a negative control stimulant. Cells were cultivated for 9 days until nucleic acid isolation.

### 4.3. Preparation of RNA and 3′ RNA-seq Libraries, and Next-Generation Sequencing

The cell lines were the sources of RNA preparation, which was performed using an AllPrep DNA/RNA Mini Kit (Qiagen, Hilden, Germany). RNA was obtained using on average 1 million cells and additional DNase treatment. The quality of RNA samples was checked on the TapeStation system (Agilent Technologies, Inc., Santa Clara, CA, USA). The RIN values of the RNA samples were above 9.0. A colorimetric measurement using a Qubit 2.0 assay (Thermofisher Scientific, Waltham, MA, USA) was implemented to calculate the recommended amount of RNA sample to be normalized for library production using the QuantSeq 3 mRNA-seq Library Prep Kit (FWD) for Illumina (Lexogen GmbH, Vienna, Austria). The protocol of library preparation comprised first-strand cDNA synthesis with oligo(dT) priming, RNA removal, second-strand synthesis by random priming, and library enrichment by PCR using the manufacturer’s recommendations, including purification steps. The quality of the produced 3′ mRNA-seq libraries was evaluated using a TapeStation system (Agilent Technologies, Inc., Santa Clara, CA, USA). The mean fragment size of the obtained libraries was 257 bp (libraries of 235 to 309 bp). Sixteen 3′ mRNA-seq libraries were pooled for the total molarity of 10 nM and were submitted for NGS comprising 50 cycles of single-read sequencing on the HiSeq Illumina platform.

### 4.4. Trimming, Filtering, Quantification, and Mapping of Demultiplexed NGS Reads, and Differential Analysis

Firstly, raw reads were checked for quality purposes with FastQC software (Babraham Bioinformatics, Boston, MA, USA). Then, reads were filtered to remove short ones (minimal read length set to 36) and those of low quality (Phred quality under 20). Adapter sequences were also removed (Flexbar software) [57]. After the filtration procedure, mapping to the EquCab 3.0 genome (GCA_002863925.1) was utilized with the use of Tophat2 software on default settings [58]. The successfully mapped reads were then counted in the ensemble annotation file (gtf file version 101) with the use of htseq-count software [59].

Differentially expressed genes (DEGs) were estimated using DESeq2 software [60] using default parameters. Then, genes with *p*-adjusted < 0.05 (Benjamini–Hochberg *p*-value adjustment) and fold change ≥ 1 were regarded as differentially expressed and used for further analysis.

### 4.5. DEGs’ Functional Annotation in KEGG Pathways

In order to retrieve the biological context of the stimulatory effects of the epigenetic agents, the DEGs between biological groups were the subject of overrepresentation tests in pathways of Kyoto Encyclopedia of Genes and Genomes (KEGG) using DAVID functional annotation tools [61]. Venn diagrams [62] and heat maps [63] were produced in order to show the magnitude of differential expression for each group of genes.

### 4.6. Real-Time PCR

First, 200 ng of total RNA was reverse-transcribed using a High-Capacity cDNA Reverse Transcription Kit (Thermofisher Scientific, Waltham, MA, USA). RT-PCR primers were designed for regions spanning at least one intron or covering an exon junction using Primer-BLAST (Table S5). Real-time PCR was performed in triplicate for each cDNA sample with a total volume of 10 μL for each sample using Sensitive RT HS-PCR Mix EvaGreen (A&A Biotechnology, Gdynia, Poland). It was run for 45 cycles with the annealing Ta of 60 °C in a QuantStudio 7 Flex System (Thermofisher Scientific, Waltham, MA, USA). Quantification of mRNA levels was performed using the comparative ΔΔCT method [64]. The relative mRNA abundances of *RPLP0* and *SDHA* genes were applied as endogenous controls. Outliers were filtered out using Grubb’s tests. The normality of the distribution was tested using the Shapiro–Wilk test, and the differences between four groups of cells were calculated as RQ values on the basis of Mann–Whitney U tests. Violin plots generated with the help of Orange data mining tools [63] were used to show the differences in fold change.

## 5. Conclusions and Future Goals

Although TSA-mediated epigenomic modulation of the ex vivo expanded horse chondrocyte cell lines has been mechanistically proven to bring about the diminishment in the expression of a broad spectrum of genes related to chondrogenic differentiation, comprehensively identifying genomic and epigenomic signatures in in vitro cultured equine chondrocytes that have been epigenetically transformed by exposure to TSA and/or 5-AZA-dc might still be indispensable to provide genetically and epigenetically reprogrammable/dedifferentiable cell lines of adult cartilage-derived somatic cells intended for future goals.

The latter is directed at ex situ study to protect a completely new source of epigenetically modified nuclear donor cells (NDCs) for the purposes of generating cloned horse embryos, conceptuses, and offspring by somatic cell nuclear transfer (SCNT).

To the best of our knowledge, the conceptualization of recognizing epigenetic plasticity and reprogrammability of TSA- and/or 5-AZA-dc-modulated adult chondrocytes in equine SCNT-generated embryos has been developed for the first time. This entirely novel approach might turn out to be a research model reliable and feasible for SCNT-mediated production of monogenetic and monosexual specimens not only in horses but also in other mammalian species.

In summary, determining the suitability of the above-indicated research model seems to be an excellent solution inevitable in regenerative medicine and reconstructive surgery of the equine chondroskeletal system.

## Figures and Tables

**Table 1:** Results of the alignment of 3’ mRNA-seq reads against the EquCab 3.0 reference sequence of the horse genome.

Group	3rd Passage Chondrocyte Stimulation	3’ mRNA-Seq Library	Total Reads after Filtering	Number of Uniquely Mapped Reads
I	5-AZA-dc ^1^	2aza-1	3,114,172	2,541,510 (81.6%)
I	5-AZA-dc	3aza-1	2,685,011	2,216,276 (82.5%)
I	5-AZA-dc	4/DEO	2,332,147	1,913,713 (82.1%)
I	5-AZA-dc	6/DEO	2,603,715	2,137,736 (82.1%)
II	5-AZA-dc+TSA ^2^	3tsaza-1	3,490,960	2,862,115 (82.0%)
II	5-AZA-dc+TSA	2tsaaza-1	2,915,748	2,336,842 (80.1%)
II	5-AZA-dc+TSA	4/Deo+TSA	3,091,055	2,548,432 (82.4%)
II	5-AZA-dc+TSA	6/DEO+TSA	2,821,472	2,329,453 (82.6%)
III	Control	2k-1	2,361,866	1,954,158 (82.7%)
III	Control	3k-1	3,062,350	2,511,373 (82.0%)
III	Control	4/K	3,312,867	2,732,432 (82.5%)
III	Control	6/K	3,207,522	2,627,829 (81.9%)
IV	TSA	3tsa-1	3,699,592	3,054,305 (82.6%)
IV	TSA	2tsa-1	2,753,414	2,261,816 (82.1%)
IV	TSA	4/TSA	3,158,031	2,620,954 (83.0%)
IV	TSA	6/TSA	2,858,574	2,361,644 (82.6%)

^1^ 5-AZA-dc concentration of 25 µg/mL. ^2^ TSA concentration of 0.25 µg/mL.

## Data Availability

Sequence Read Archive (SRA): Bioproject Number PRJNA868898.

## Supplementary Materials

The following supporting information can be downloaded at: https://www.mdpi.com/article/10.3390/ijms232113168/s1.
