# Supplementary material for: Trichostatin A-Mediated Epigenetic Modulation Predominantly Triggers Transcriptomic Alterations in the Ex Vivo Expanded Equine Chondrocytes
Source: Int J Mol Sci. 2022 Oct 29;23(21):13168. doi: 10.3390/ijms232113168 (PMC9655705; doi:10.3390/ijms232113168)
Supplement: Supplementary file 1 [file ijms-23-13168-s001.zip › Supplementary Figures.pdf]

Results of validation step for 42 genes found to be differentially expressed based on NGS experiment.

Figures S1 to S42 include results of real-time PCR of 42 genes differentially expressed between I vs IV (5-AZA-dc versus TSA), I vs II (5-AZA-dc versus 5-AZA-dc+TSA), II vs III (5-AZA-dc+TSA versus control group) and III vs IV (control group versus TSA). Values of fold change of expression for each gene are located on the y-axis.

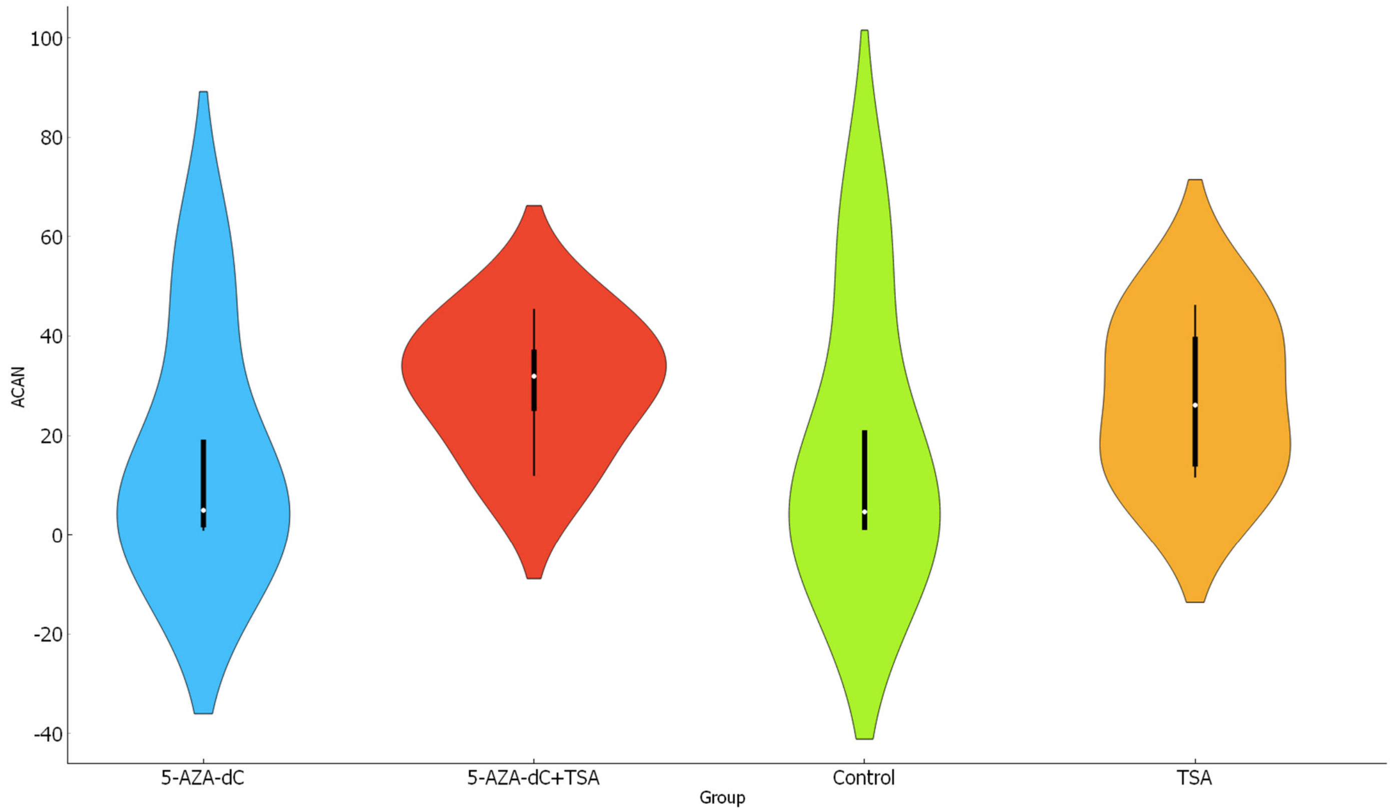

Figure S1. Results of real-time PCR analysis of ACAN gene in chondrocytes dependently on applied stimulation: 5-AZA-dc, 5-AZA-dc+TSA, control and TSA. *p-value < 0.05, ** p-value < 0.01, ns - p > 0.05

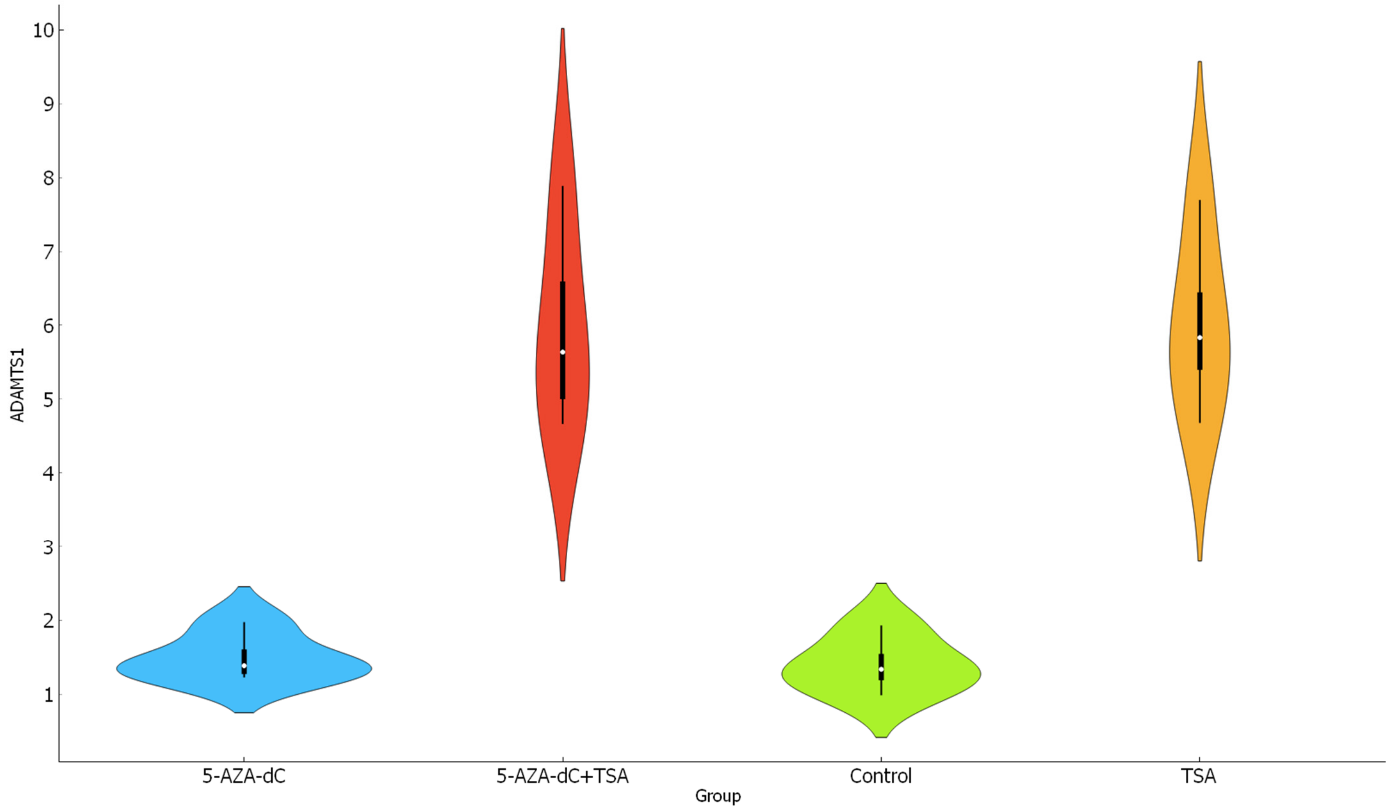

Figure S2. Results of real-time PCR analysis of ADAMTS1 gene in chondrocytes dependently on applied stimulation: 5-AZA-dc, 5-AZA-dc+TSA, control and TSA. *p-value < 0.05, ** p-value < 0.01, ns - p > 0.05

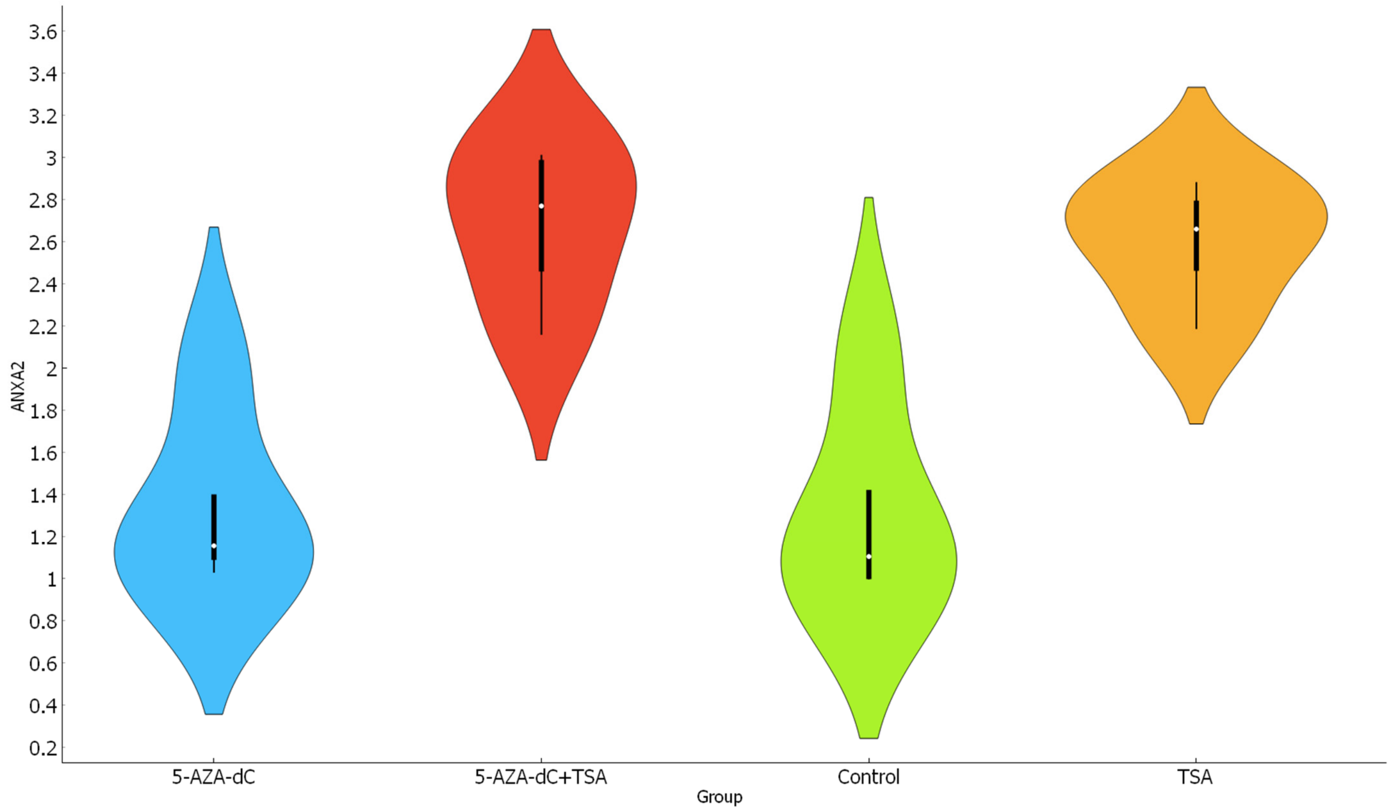

Figure S3. Results of real-time PCR analysis of ANXA2 gene in chondrocytes dependently on applied stimulation: 5-AZA-dc, 5-AZA-dc+TSA, control and TSA. *p-value < 0.05, ** p-value < 0.01, ns - p > 0.05

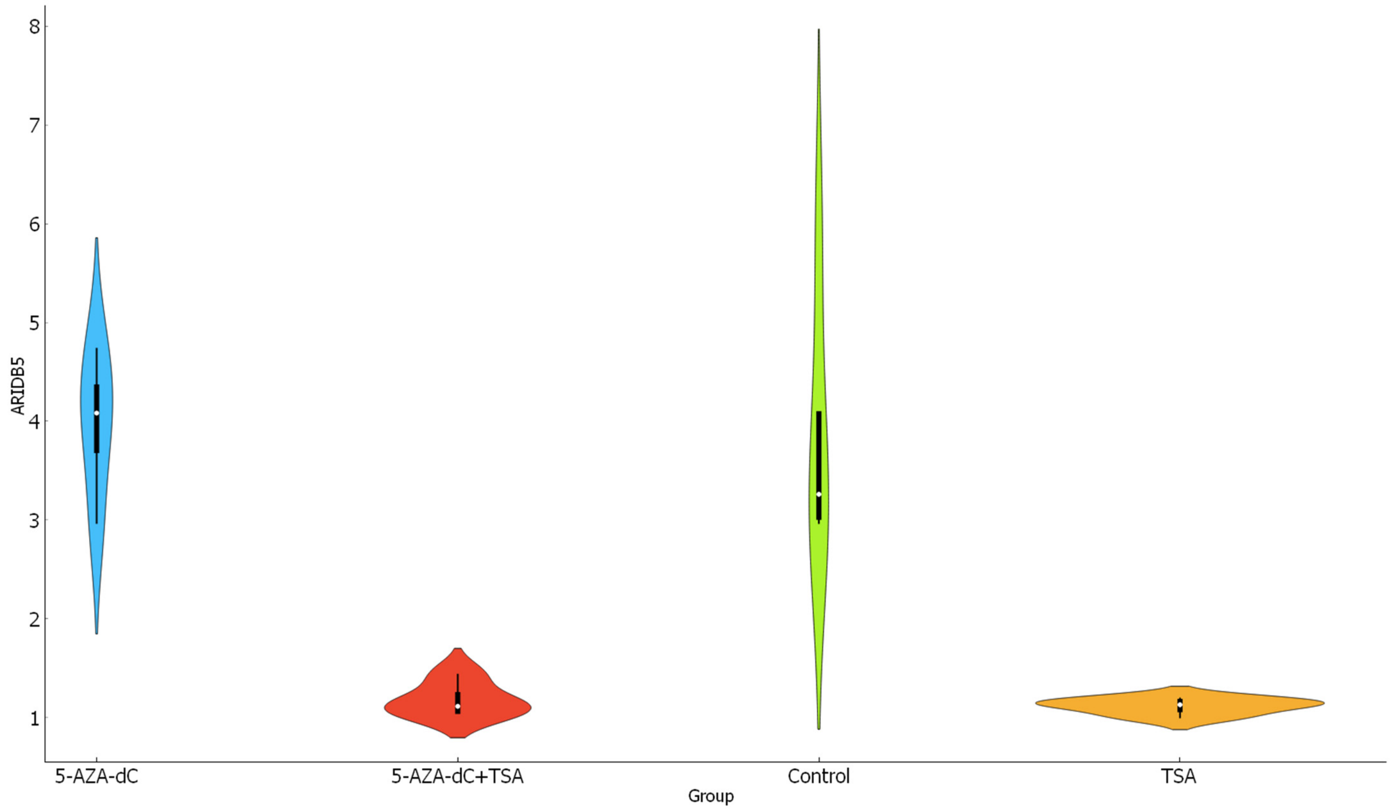

Figure S4. Results of real-time PCR analysis of ARIDB5 gene in chondrocytes dependently on applied stimulation: 5-AZA-dc, 5-AZA-dc+TSA, control and TSA. *p-value < 0.05, ** p-value < 0.01, ns - p > 0.05

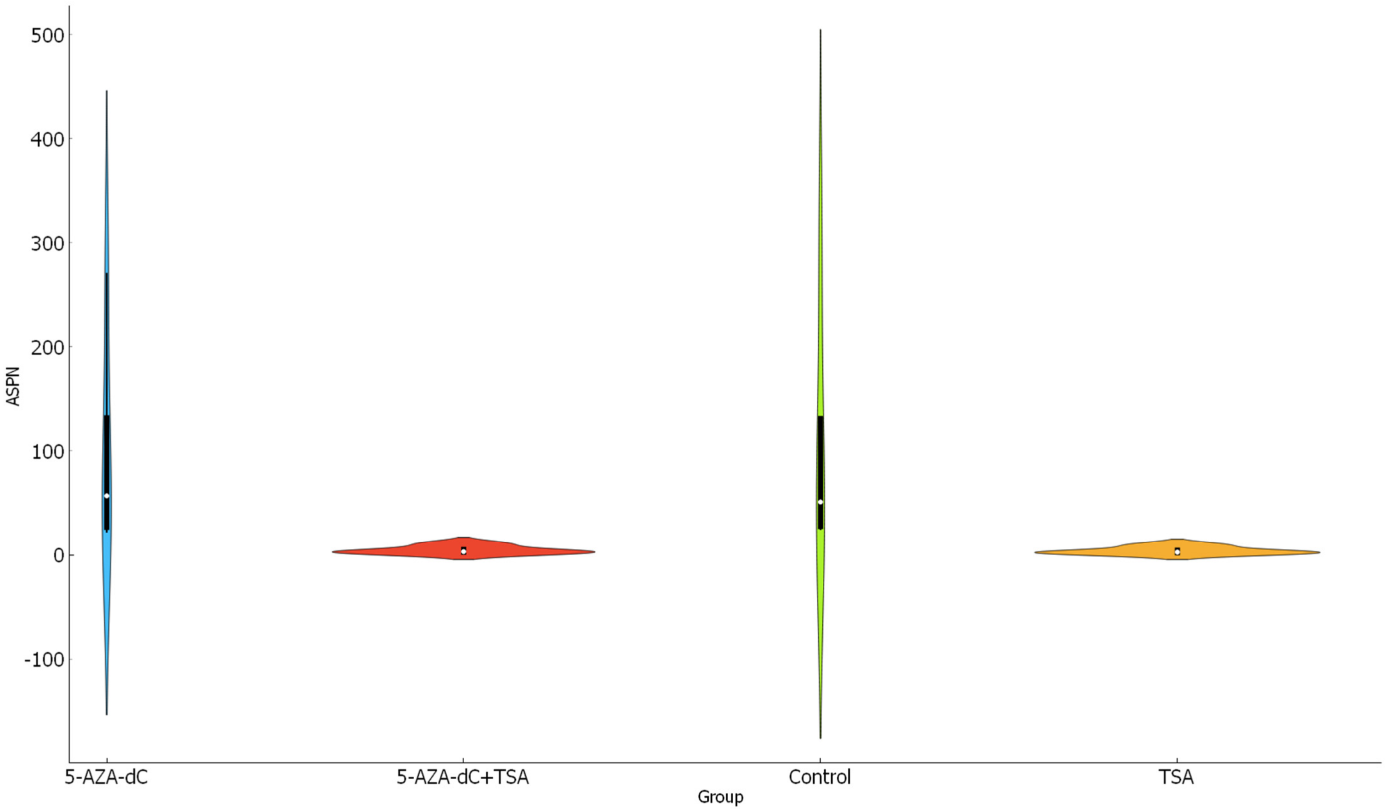

Figure S5. Results of real-time PCR analysis of ASPN gene in chondrocytes dependently on applied stimulation: 5-AZA-dc, 5-AZA-dc+TSA, control and TSA. *p-value < 0.05, ** p-value < 0.01, ns - p > 0.05

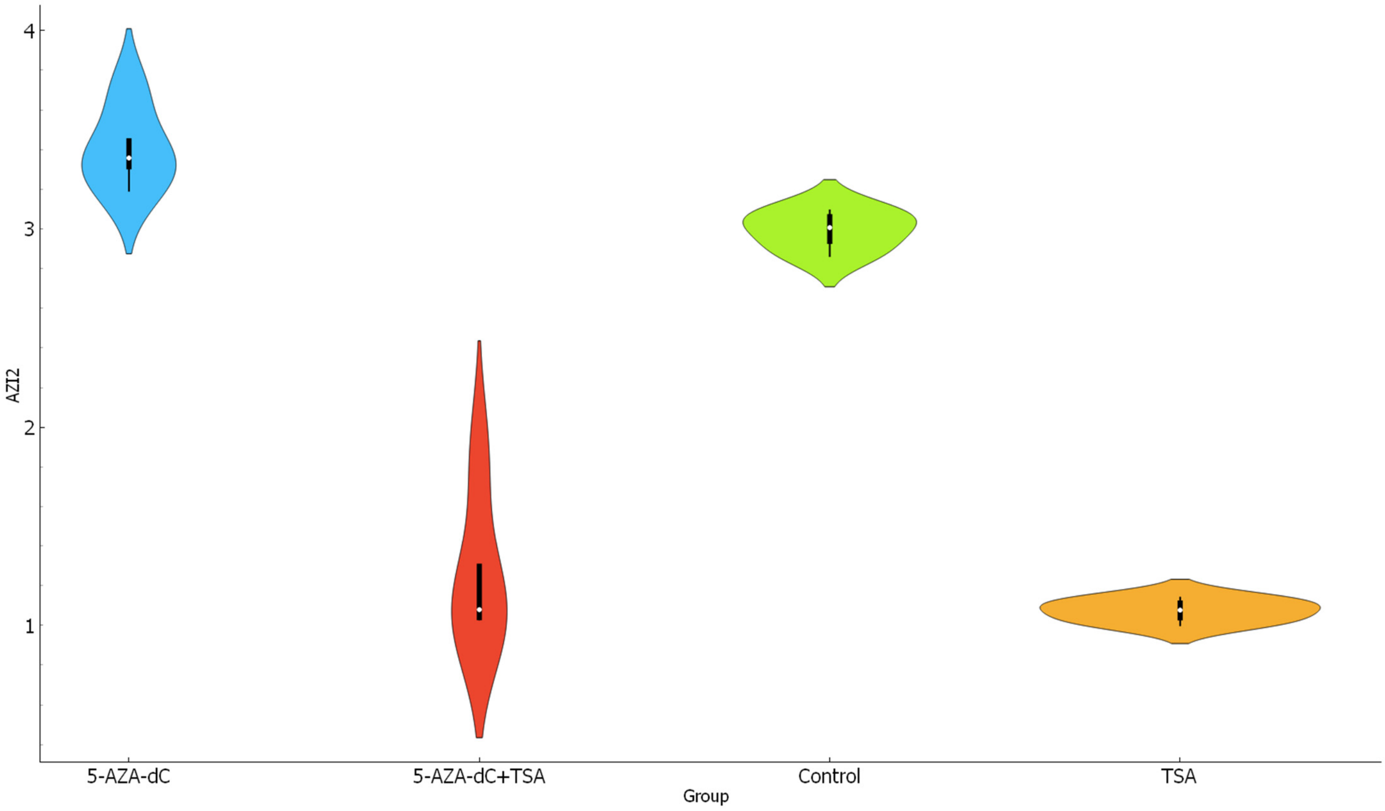

Figure S6. Results of real-time PCR analysis of AZI2 gene in chondrocytes dependently on applied stimulation: 5-AZA-dc, 5-AZA-dc+TSA, control and TSA. *p-value < 0.05, ** p-value < 0.01, ns - p > 0.05

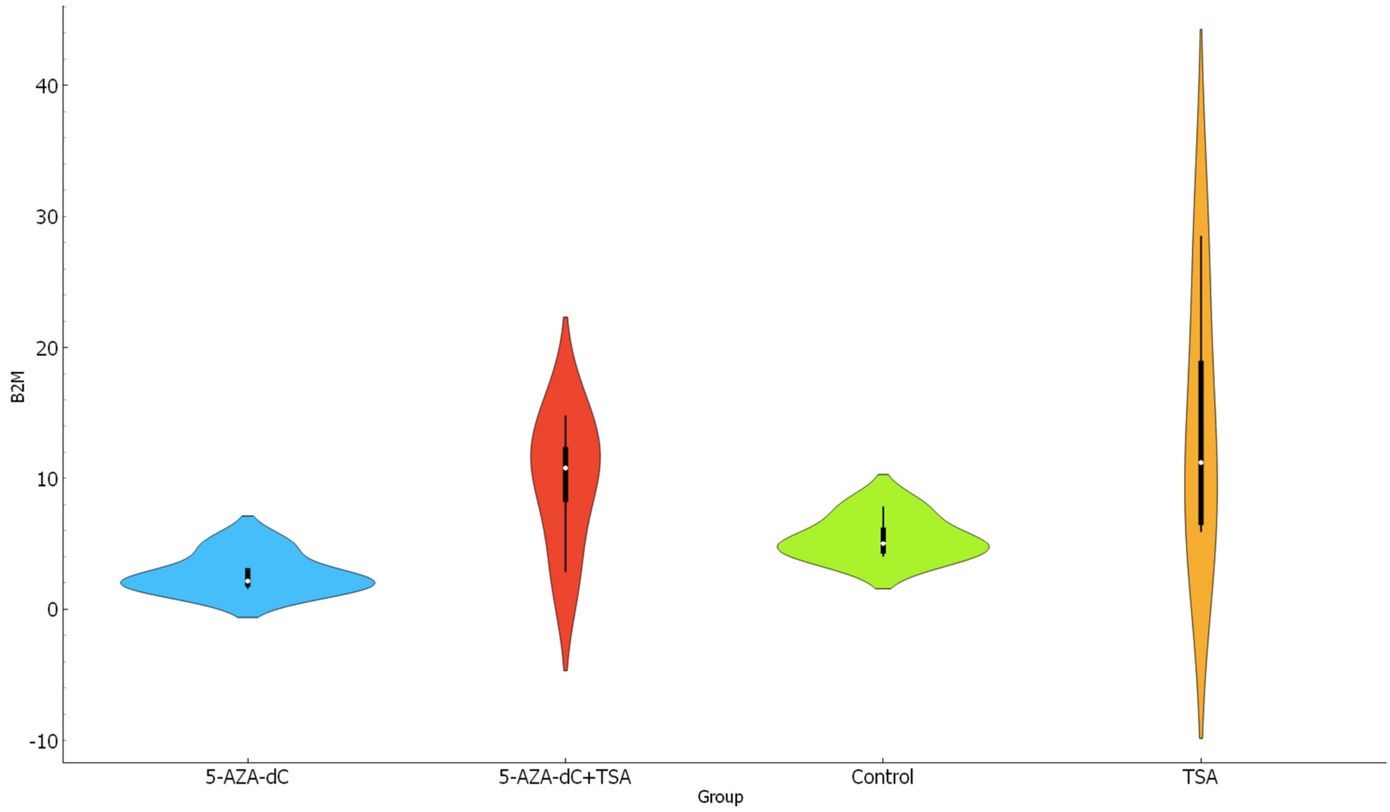

Figure S7. Results of real-time PCR analysis of B2M gene in chondrocytes dependently on applied stimulation: 5-AZA-dc, 5-AZA-dc+TSA, control and TSA. *p-value < 0.05, ** p-value < 0.01, ns - p > 0.05

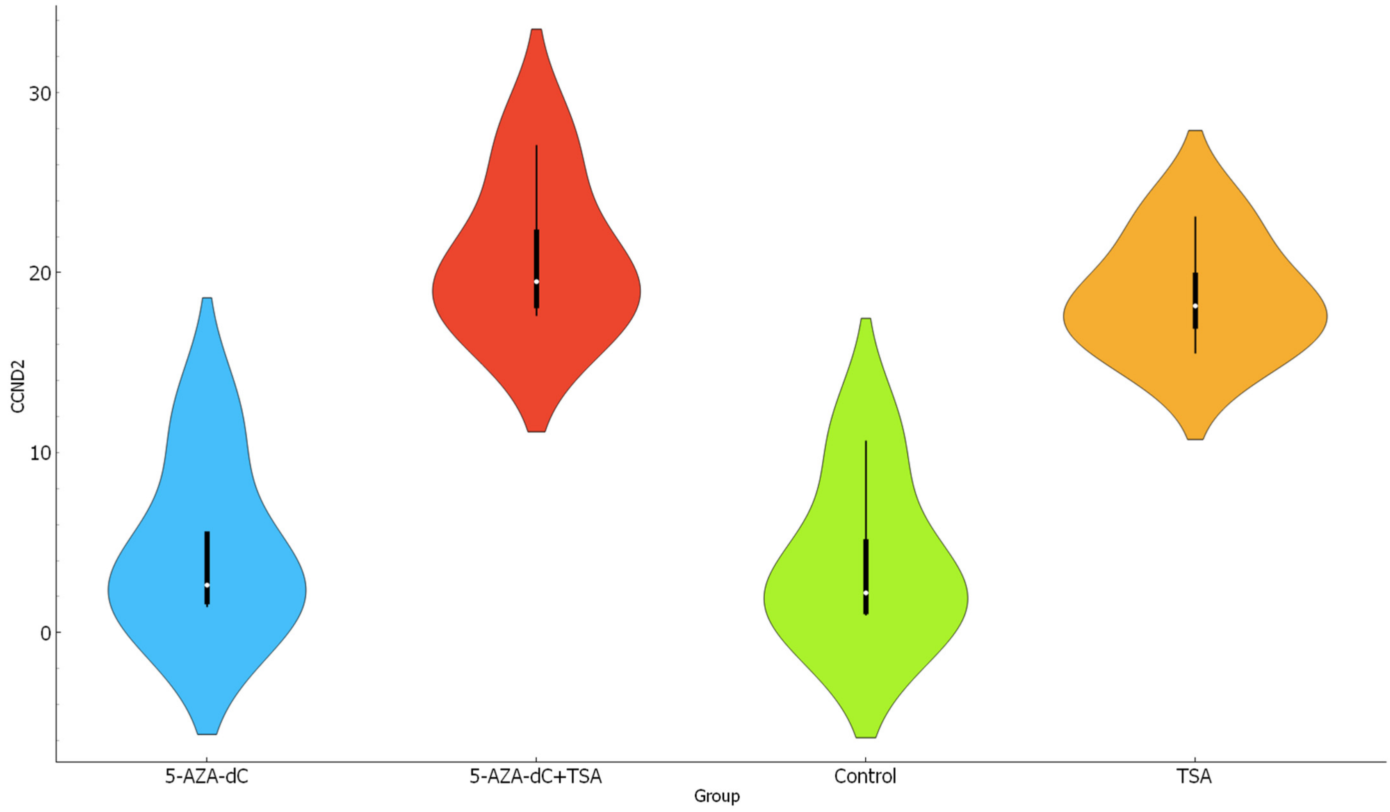

Figure S8. Results of real-time PCR analysis of CCND2 gene in chondrocytes dependently on applied stimulation: 5-AZA-dc, 5-AZA-dc+TSA, control and TSA. *p-value < 0.05, ** p-value < 0.01, ns - p > 0.05

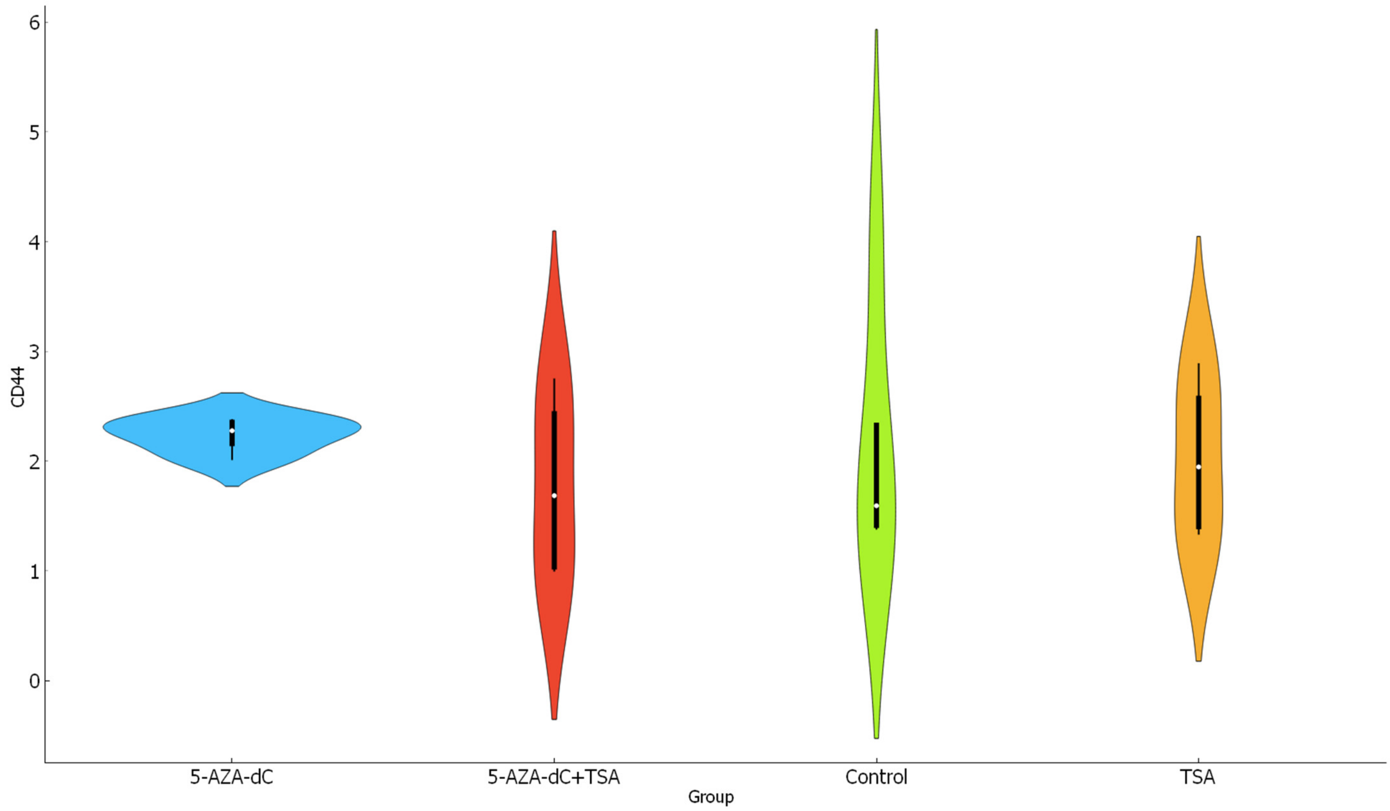

Figure S9. Results of real-time PCR analysis of CD44 gene in chondrocytes dependently on applied stimulation: 5-AZA-dc, 5-AZA-dc+TSA, control and TSA. *p-value < 0.05, ** p-value < 0.01, ns - p > 0.05

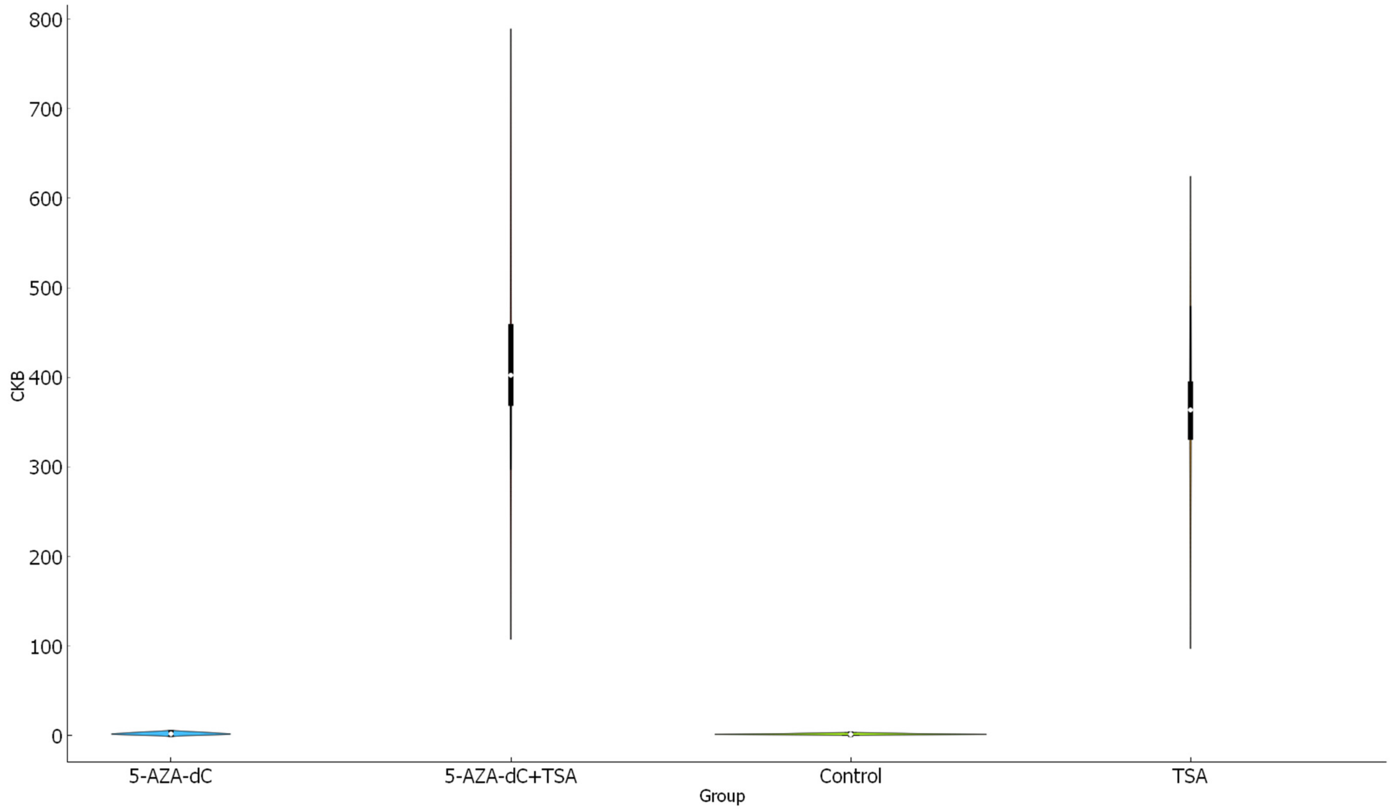

Figure S10. Results of real-time PCR analysis of CKB gene in chondrocytes dependently on applied stimulation: 5-AZA-dc, 5-AZA-dc+TSA, control and TSA. *p-value < 0.05, ** p-value < 0.01, ns - p > 0.05

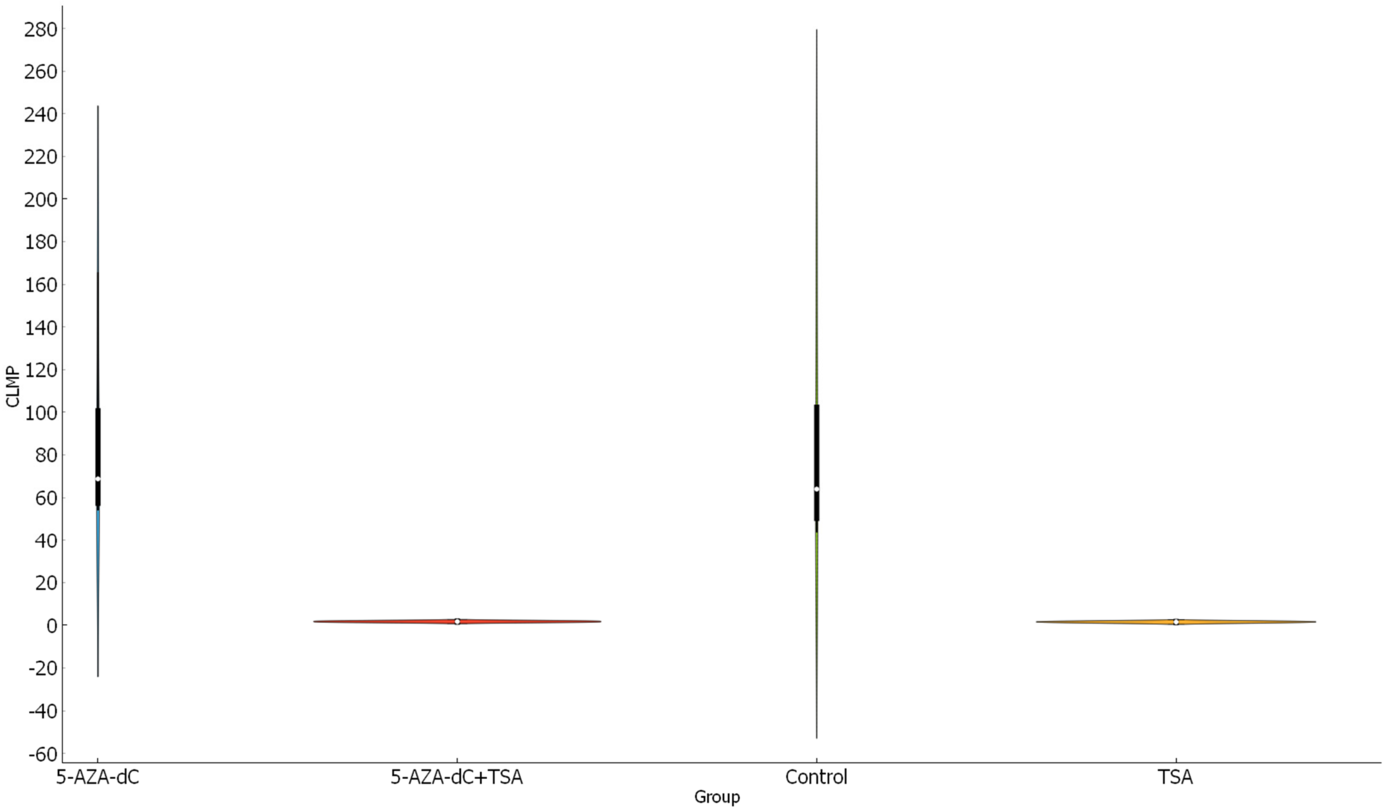

Figure S11. Results of real-time PCR analysis of CLMP gene in chondrocytes dependently on applied stimulation: 5-AZA-dc, 5-AZA-dc+TSA, control and TSA. *p-value < 0.05, ** p-value < 0.01, ns - p > 0.05

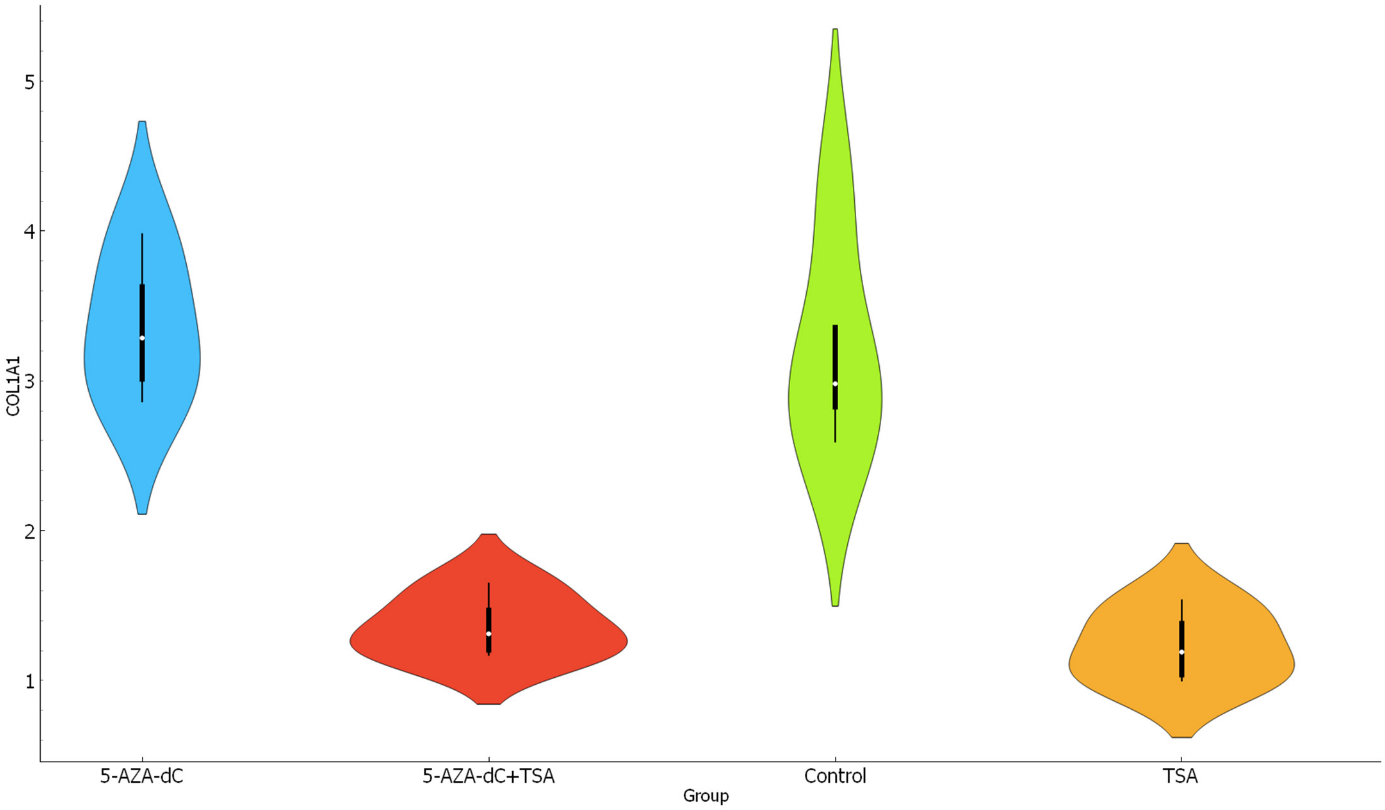

Figure S12. Results of real-time PCR analysis of COL1A1 gene in chondrocytes dependently on applied stimulation: 5-AZA-dc, 5-AZA-dc+TSA, control and TSA. *p-value < 0.05, ** p-value < 0.01, ns - p > 0.05

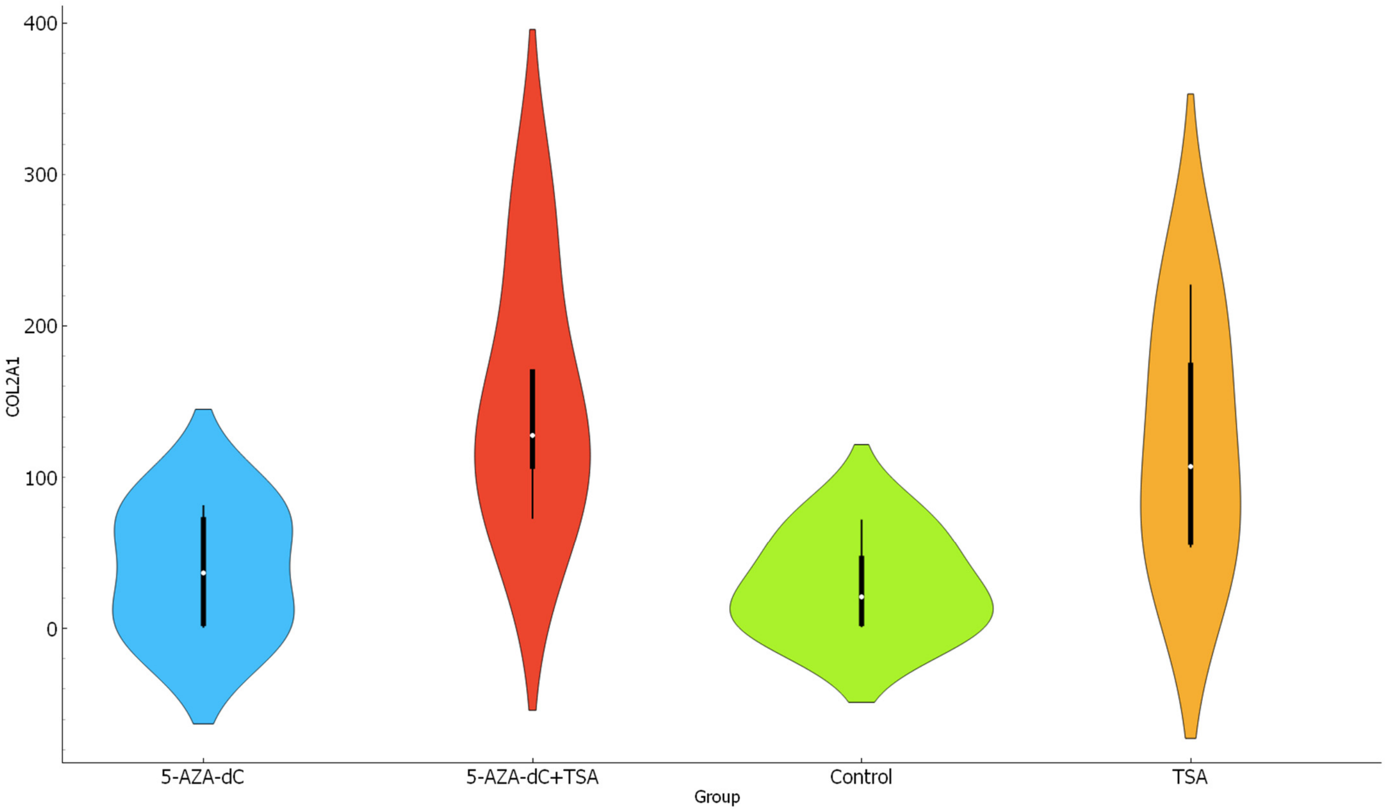

Figure S13. Results of real-time PCR analysis of COL2A1 gene in chondrocytes dependently on applied stimulation: 5-AZA-dc, 5-AZA-dc+TSA, control and TSA. *p-value < 0.05, ** p-value < 0.01, ns - p > 0.05

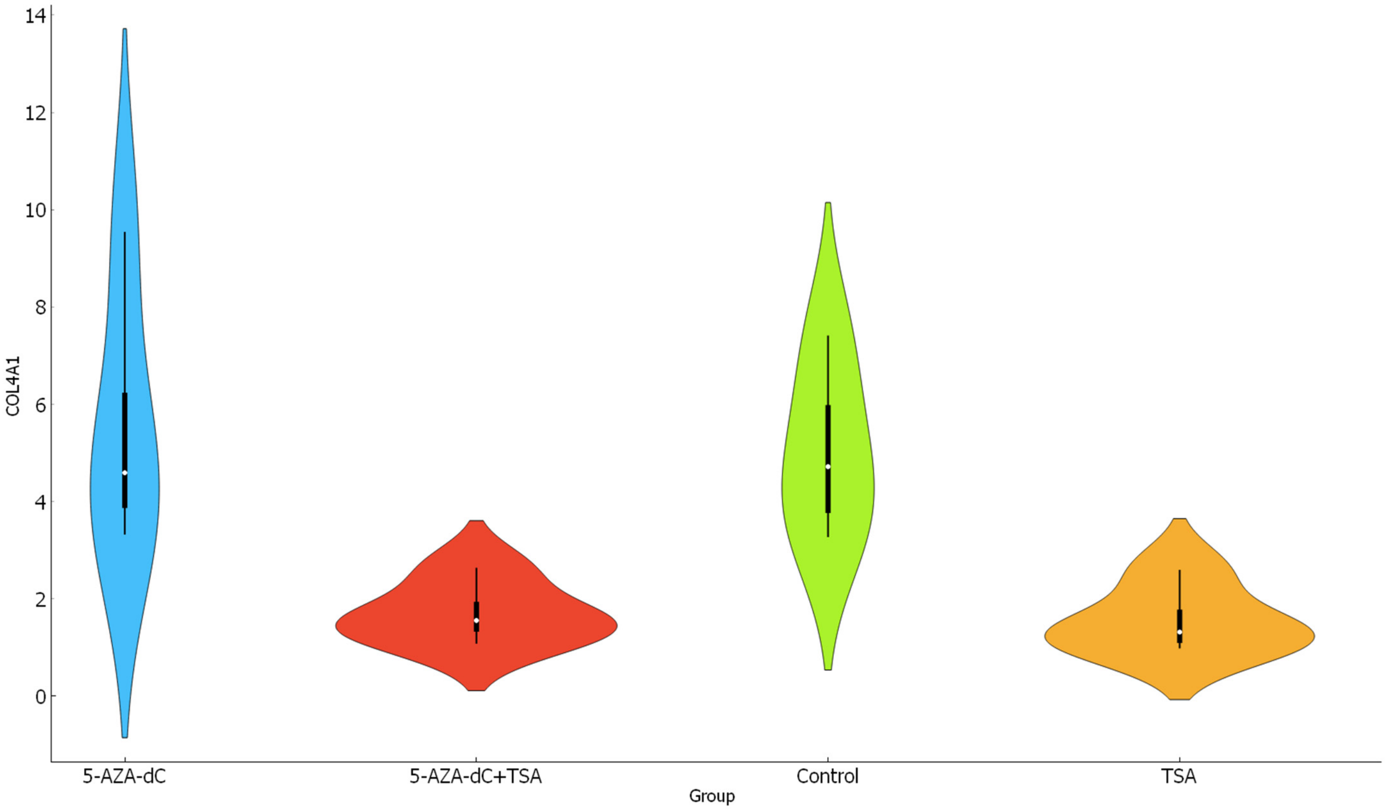

Figure S14. Results of real-time PCR analysis of COL4A1 gene in chondrocytes dependently on applied stimulation: 5-AZA-dc, 5-AZA-dc+TSA, control and TSA. *p-value < 0.05, ** p-value < 0.01, ns - p > 0.05

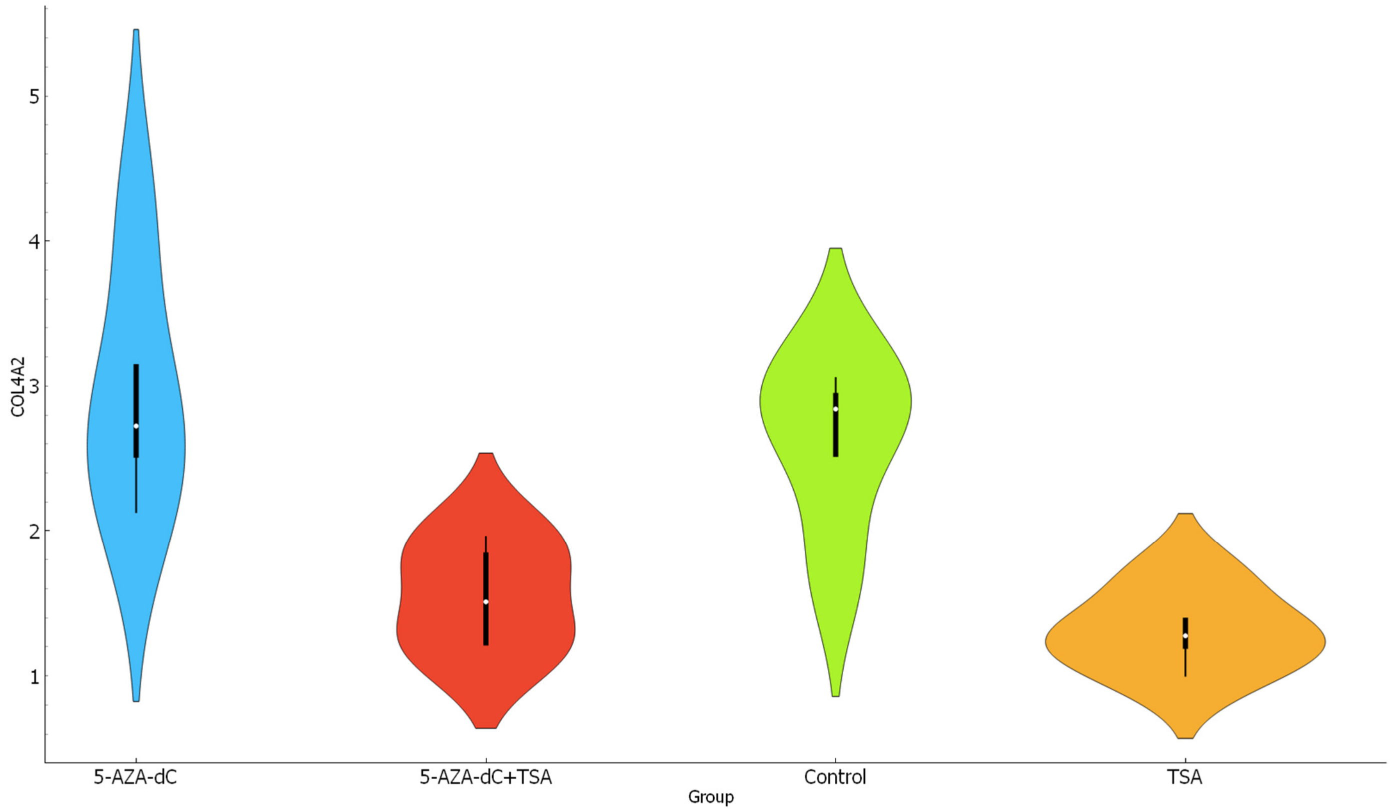

Figure S15. Results of real-time PCR analysis of COL4A2 gene in chondrocytes dependently on applied stimulation: 5-AZA-dc, 5-AZA-dc+TSA, control and TSA. *p-value < 0.05, ** p-value < 0.01, ns - p > 0.05

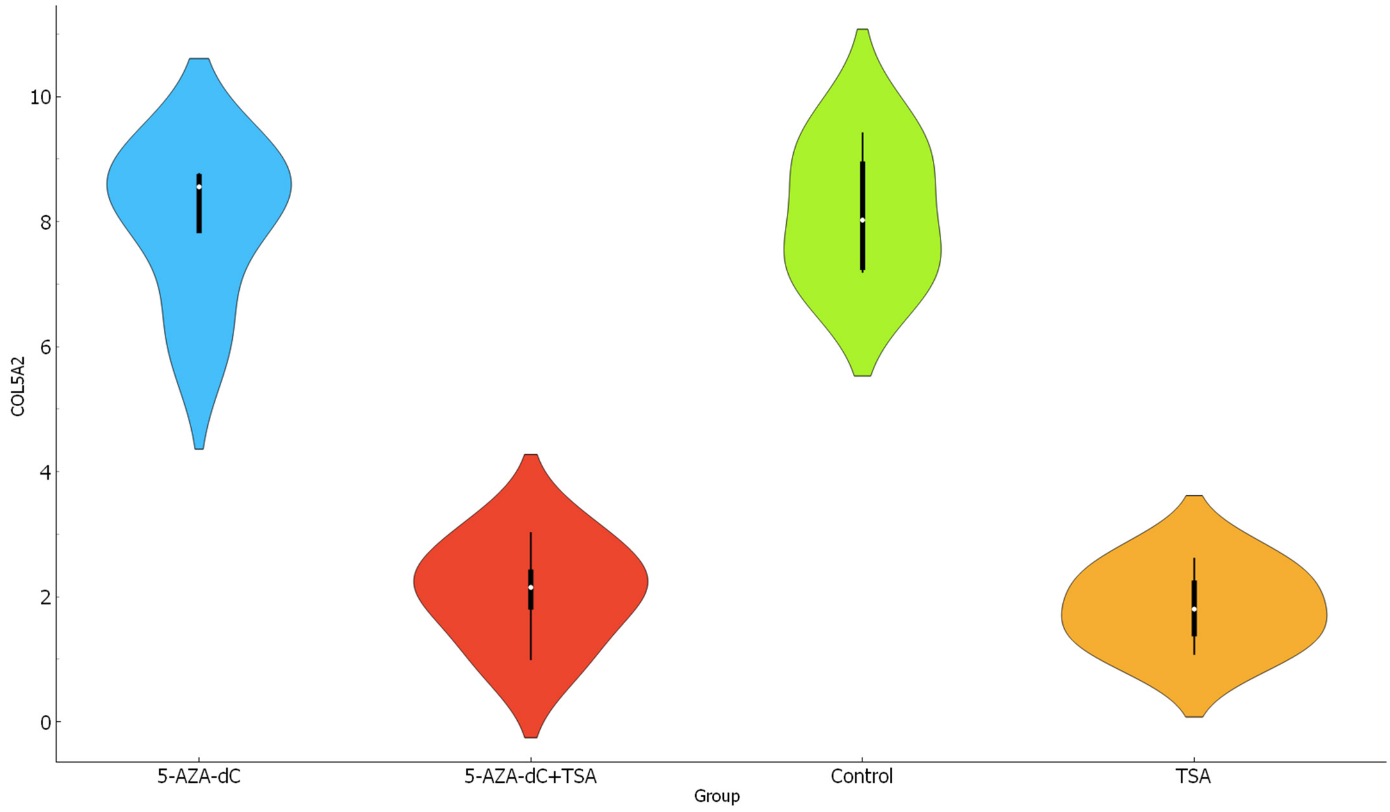

Figure S16. Results of real-time PCR analysis of COL5A2 gene in chondrocytes dependently on applied stimulation: 5-AZA-dc, 5-AZA-dc+TSA, control and TSA. *p-value < 0.05, ** p-value < 0.01, ns - p > 0.05

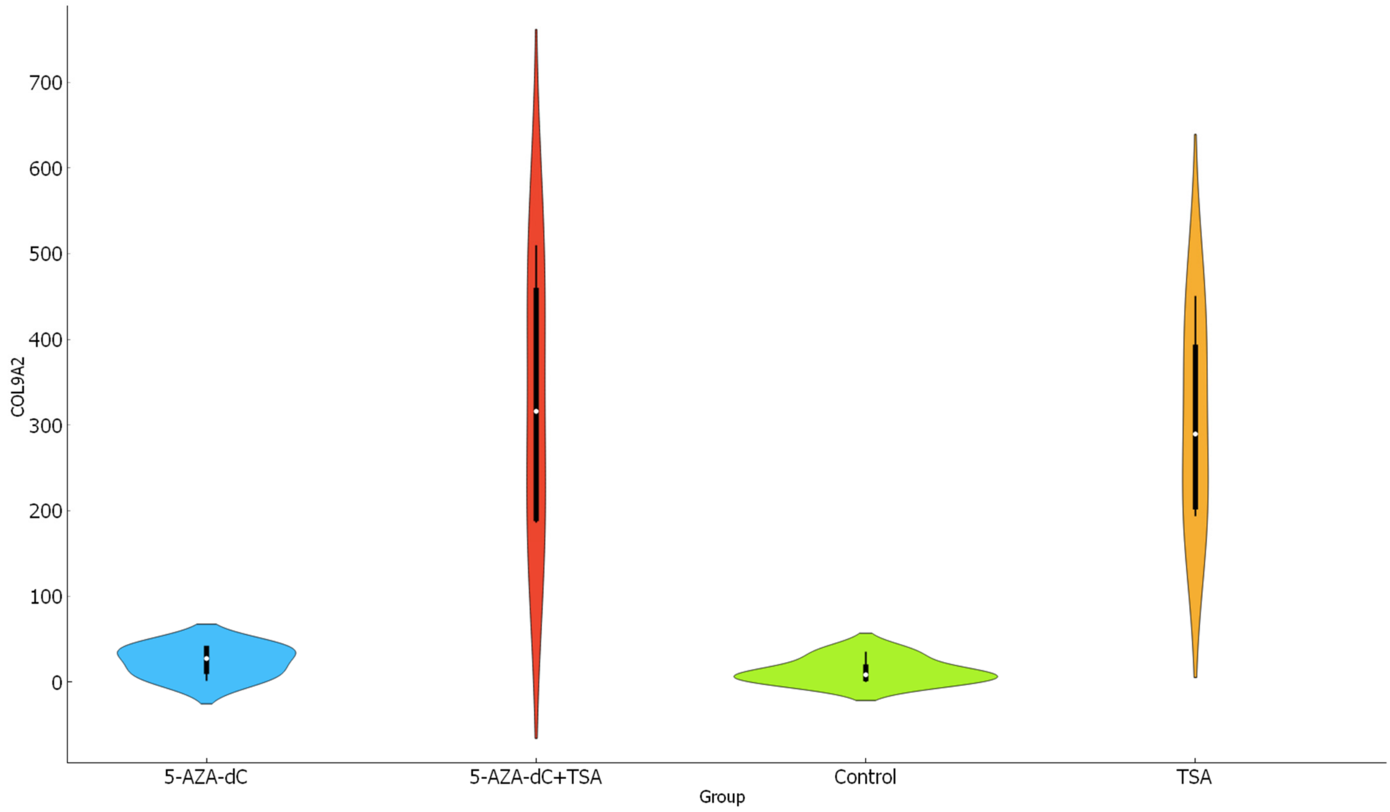

Figure S17. Results of real-time PCR analysis of COL9A2 gene in chondrocytes dependently on applied stimulation: 5-AZA-dc, 5-AZA-dc+TSA, control and TSA. *p-value < 0.05, ** p-value < 0.01, ns - p > 0.05

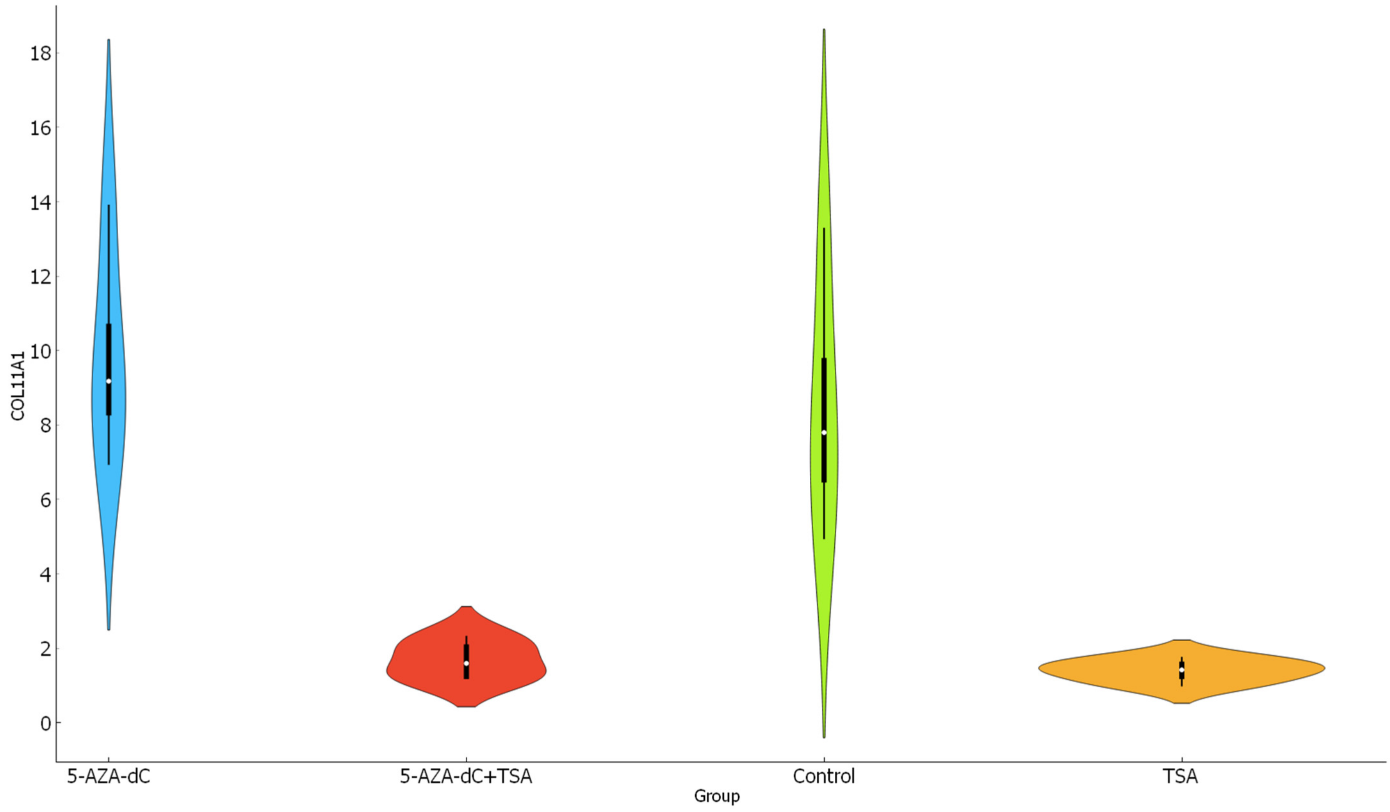

Figure S18. Results of real-time PCR analysis of COL11A1 gene in chondrocytes dependently on applied stimulation: 5-AZA-dc, 5-AZA-dc+TSA, control and TSA. *p-value < 0.05, ** p-value < 0.01, ns - p > 0.05

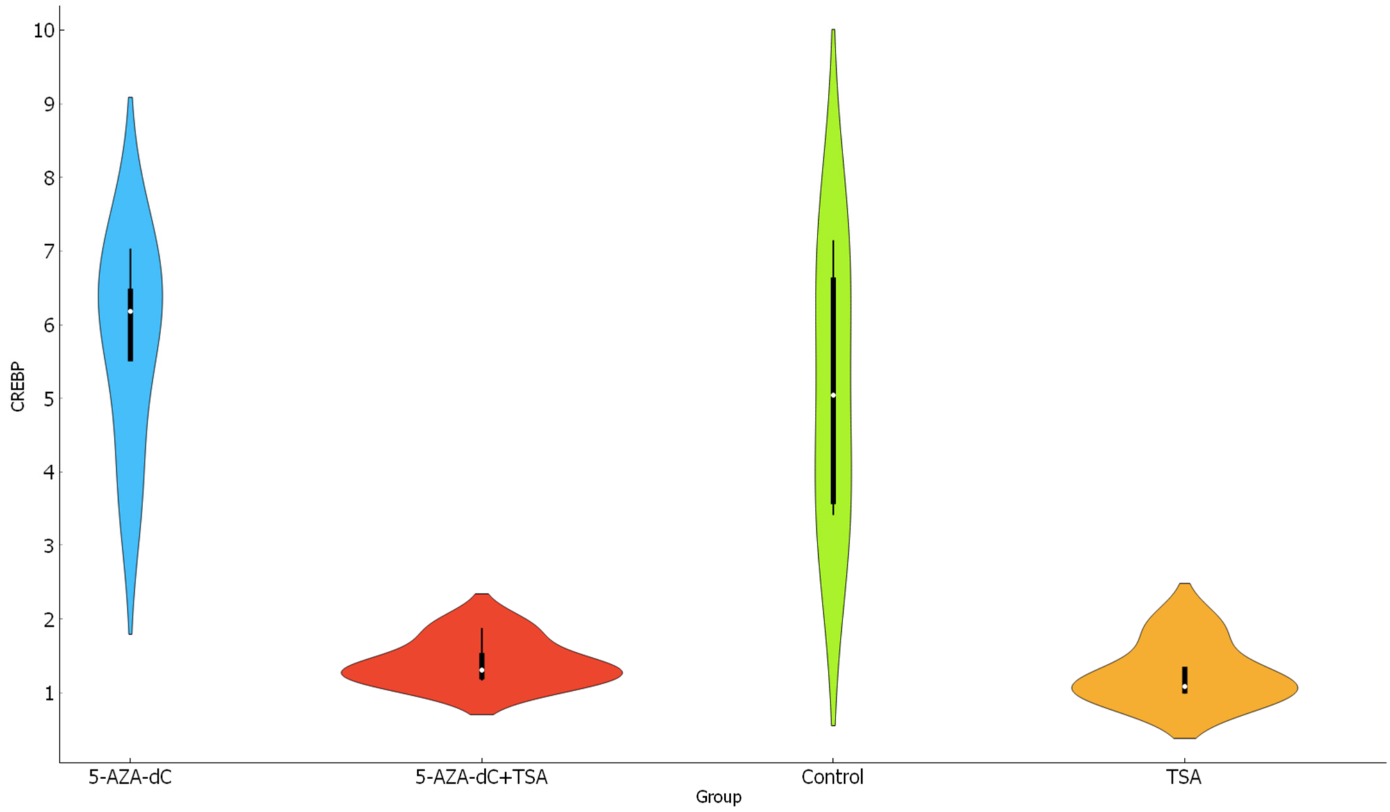

Figure S19. Results of real-time PCR analysis of CREBP gene in chondrocytes dependently on applied stimulation: 5-AZA-dc, 5-AZA-dc+TSA, control and TSA. *p-value < 0.05, ** p-value < 0.01, ns - p > 0.05

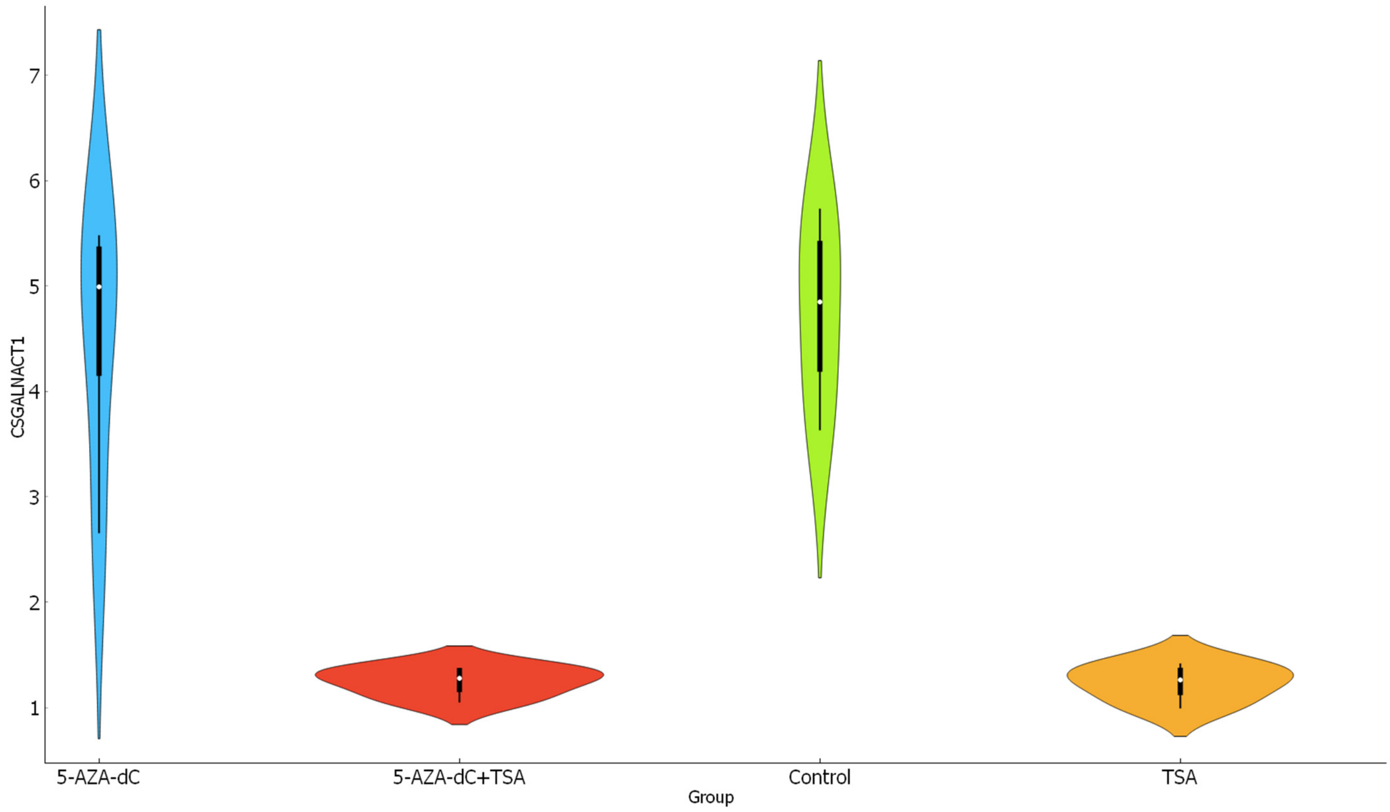

Figure S20. Results of real-time PCR analysis of CSGALNACT1 gene in chondrocytes dependently on applied stimulation: 5-AZA-dc, 5-AZA-dc+TSA, control and TSA. *p-value < 0.05, ** p-value < 0.01, ns - p > 0.05

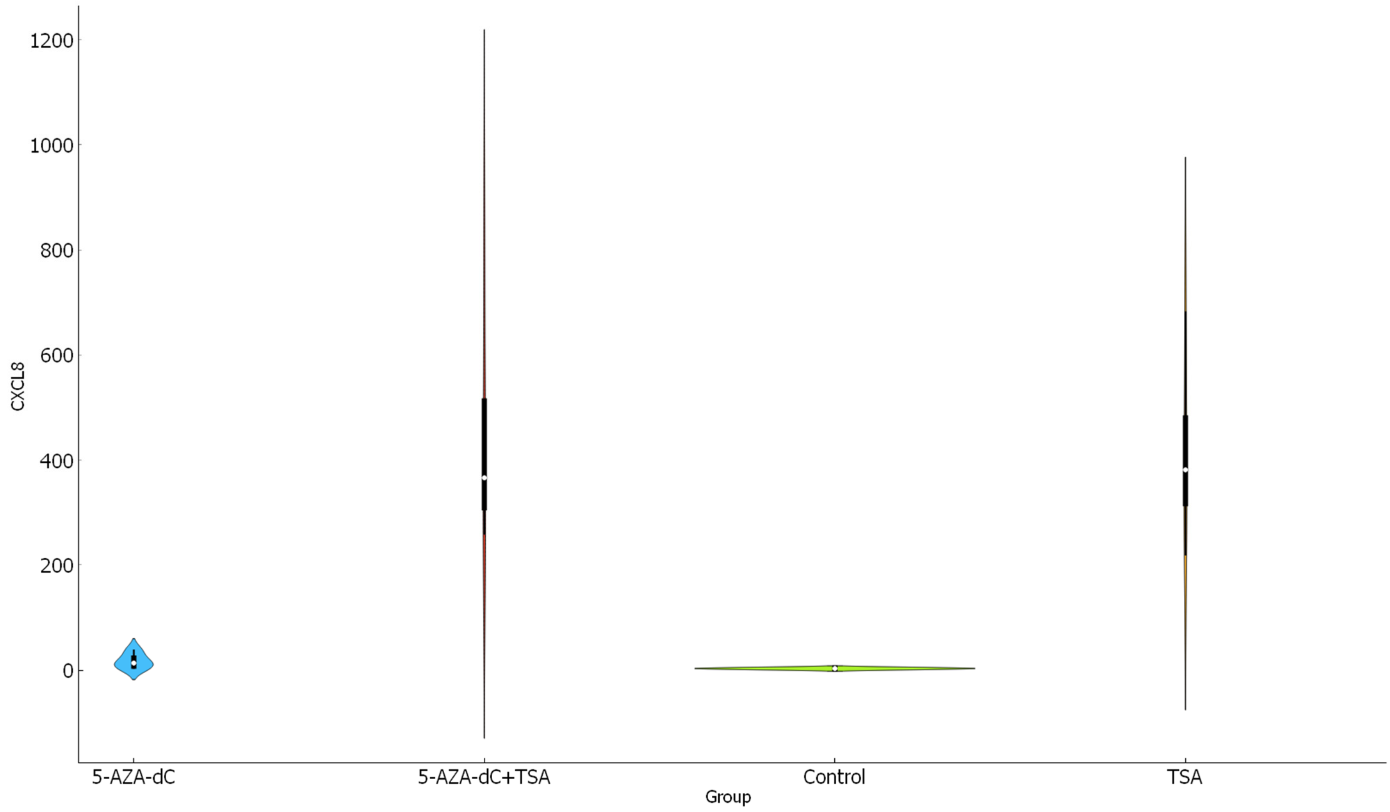

Figure S21. Results of real-time PCR analysis of CXCL8 gene in chondrocytes dependently on applied stimulation: 5-AZA-dc, 5-AZA-dc+TSA, control and TSA. *p-value < 0.05, ** p-value < 0.01, ns - p > 0.05

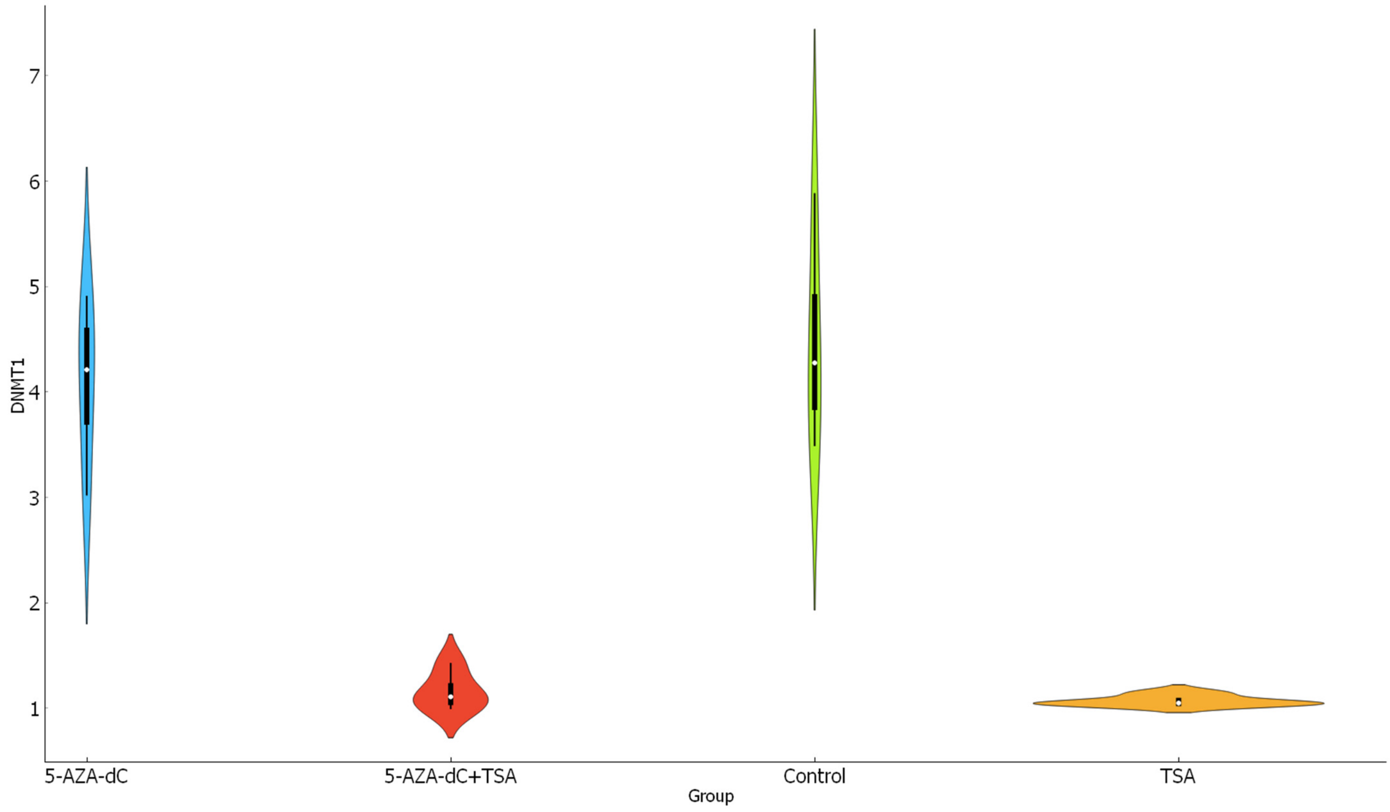

Figure S22. Results of real-time PCR analysis of DNMT1 gene in chondrocytes dependently on applied stimulation: 5-AZA-dc, 5-AZA-dc+TSA, control and TSA. *p-value < 0.05, ** p-value < 0.01, ns - p > 0.05

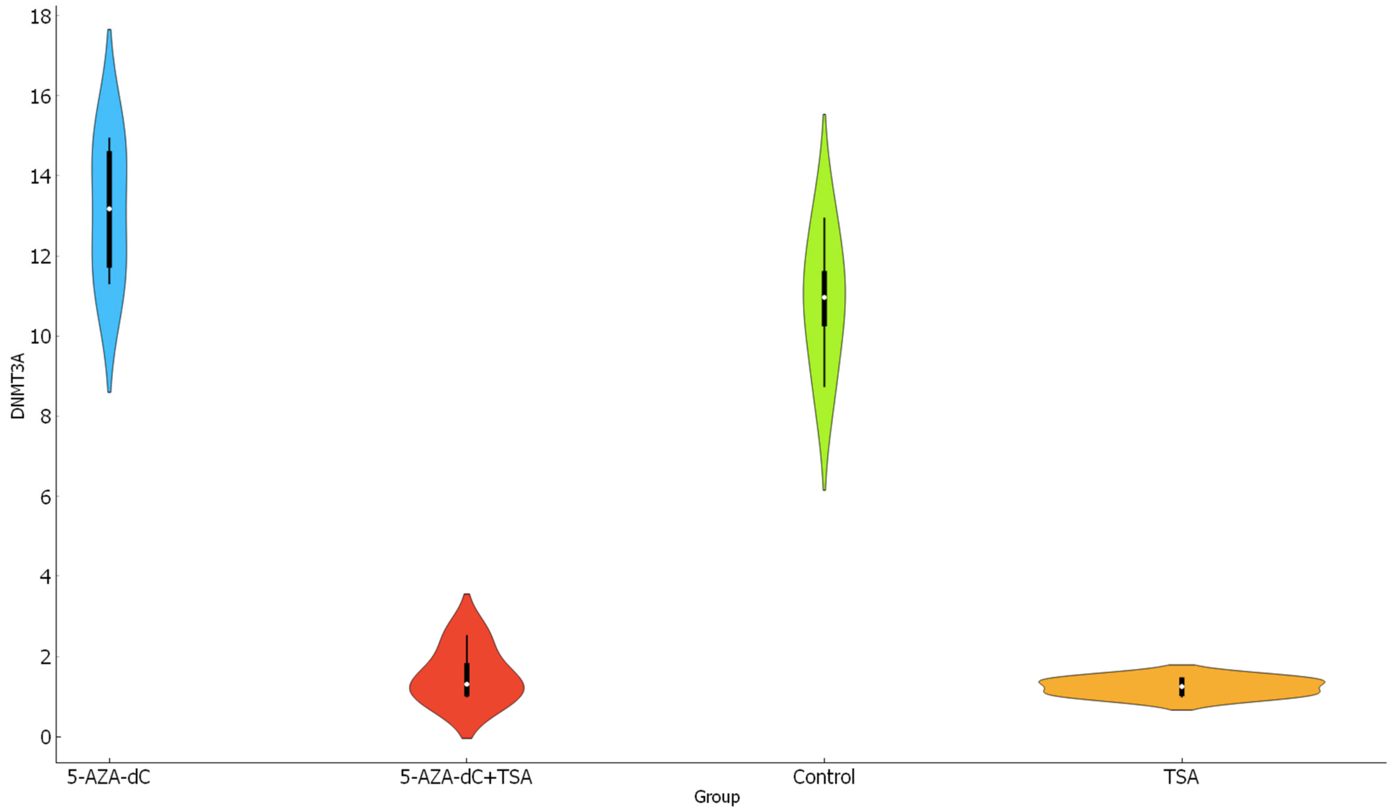

Figure S23. Results of real-time PCR analysis of DNMT3A gene in chondrocytes dependently on applied stimulation: 5-AZA-dc, 5-AZA-dc+TSA, control and TSA. *p-value < 0.05, ** p-value < 0.01, ns - p > 0.05

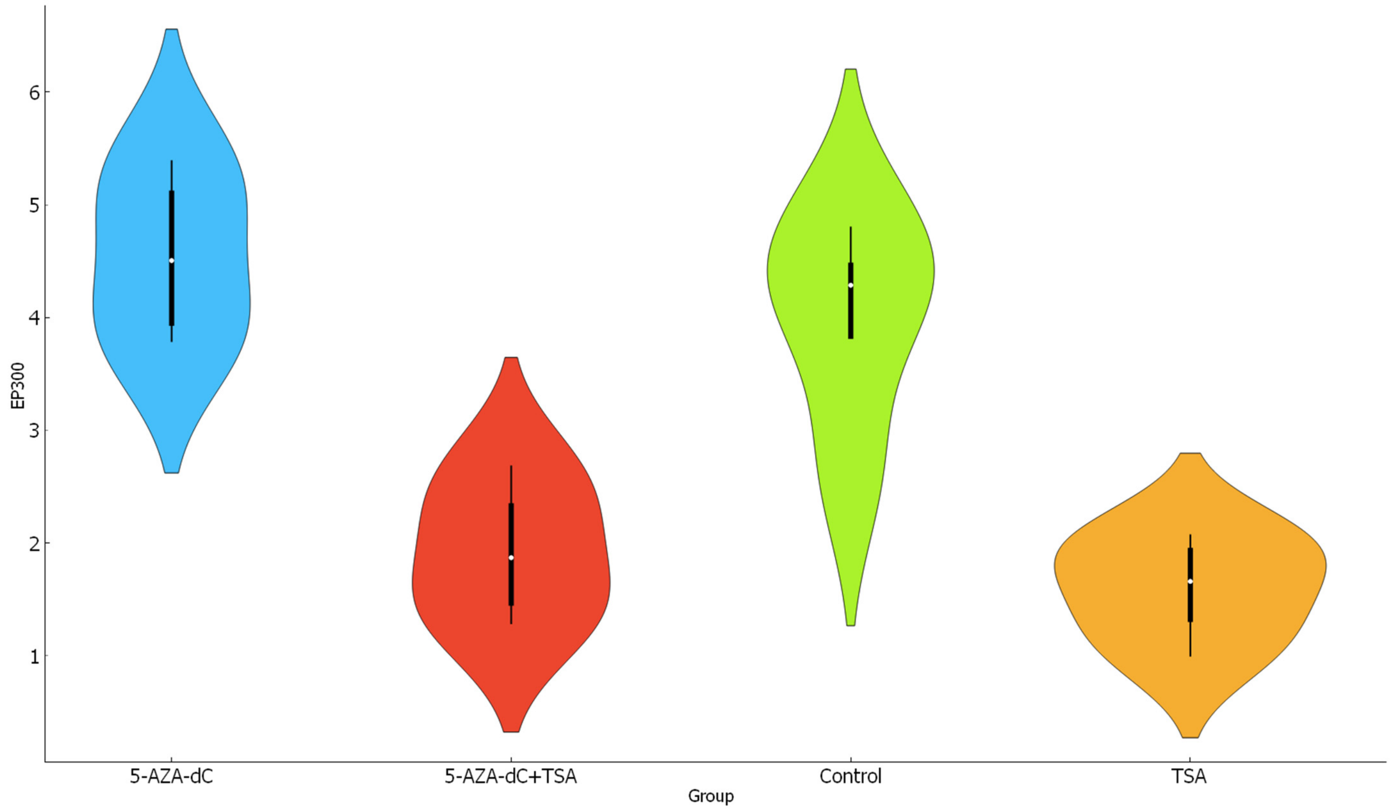

Figure S24. Results of real-time PCR analysis of EP300 gene in chondrocytes dependently on applied stimulation: 5-AZA-dc, 5-AZA-dc+TSA, control and TSA. *p-value < 0.05, ** p-value < 0.01, ns - p > 0.05

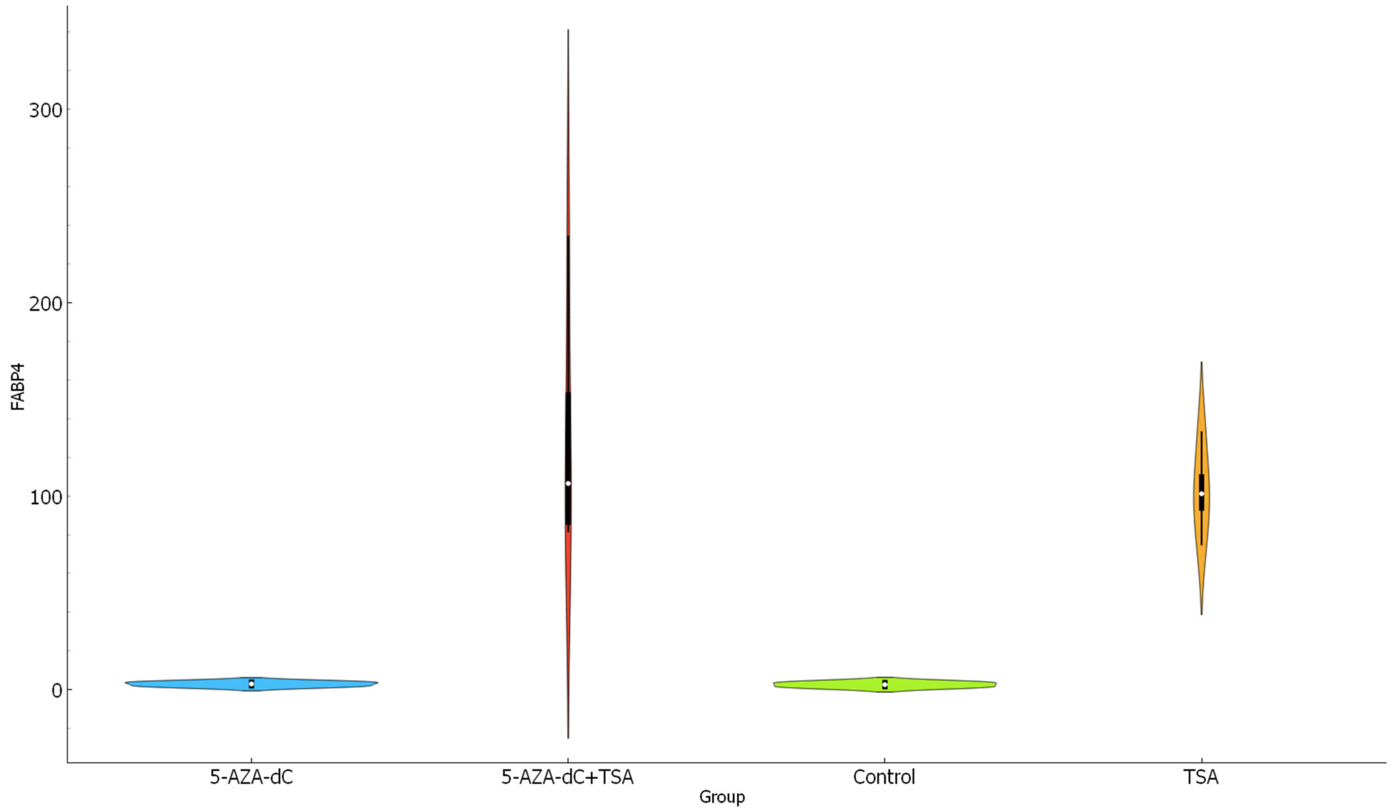

Figure S25. Results of real-time PCR analysis of FABP4 gene in chondrocytes dependently on applied stimulation: 5-AZA-dc, 5-AZA-dc+TSA, control and TSA. *p-value < 0.05, ** p-value < 0.01, ns - p > 0.05

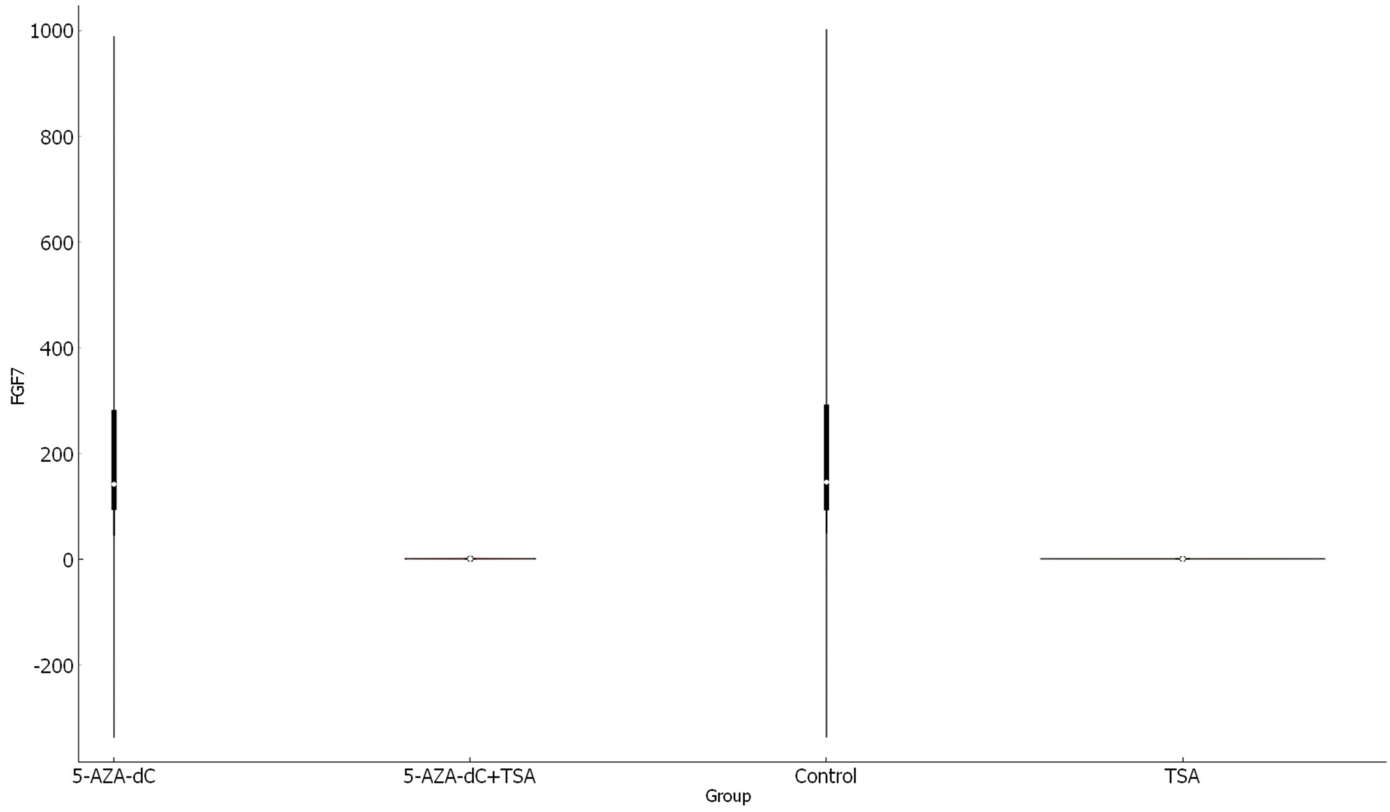

Figure S26. Results of real-time PCR analysis of FGF7 gene in chondrocytes dependently on applied stimulation: 5-AZA-dc, 5-AZA-dc+TSA, control and TSA. *p-value < 0.05, ** p-value < 0.01, ns - p > 0.05

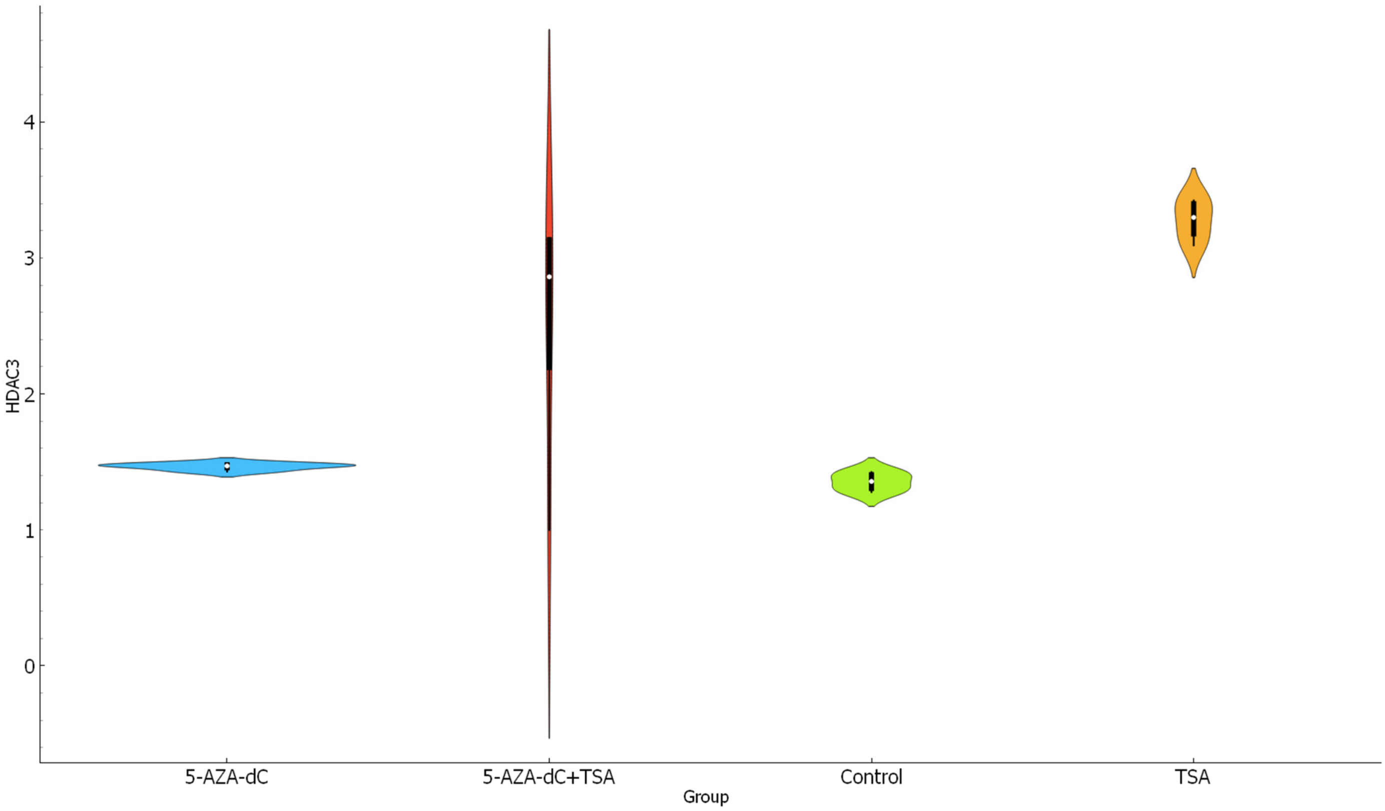

Figure S27. Results of real-time PCR analysis of HDAC3 gene in chondrocytes dependently on applied stimulation: 5-AZA-dc, 5-AZA-dc+TSA, control and TSA. *p-value < 0.05, ** p-value < 0.01, ns - p > 0.05

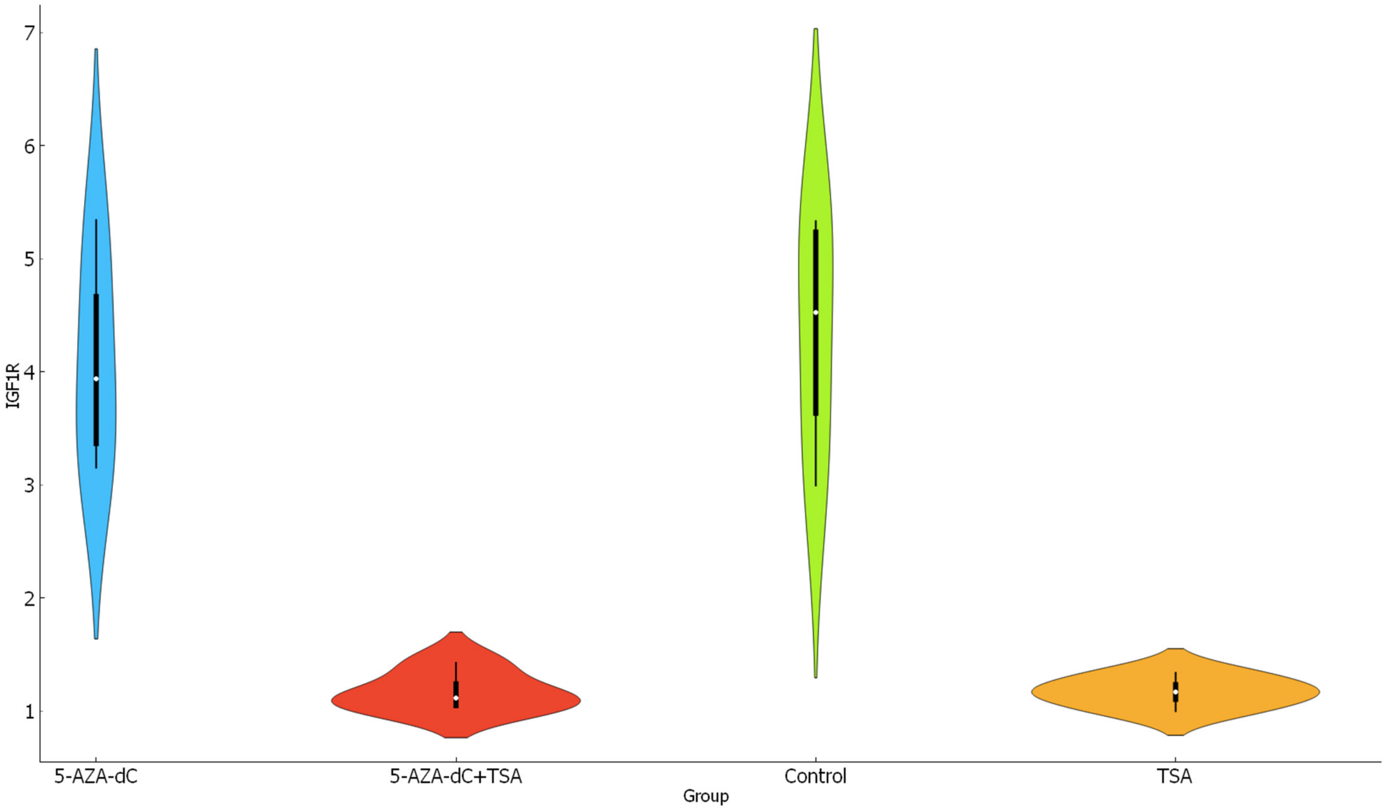

Figure S28. Results of real-time PCR analysis of IGF1R gene in chondrocytes dependently on applied stimulation: 5-AZA-dc, 5-AZA-dc+TSA, control and TSA. *p-value < 0.05, ** p-value < 0.01, ns - p > 0.05

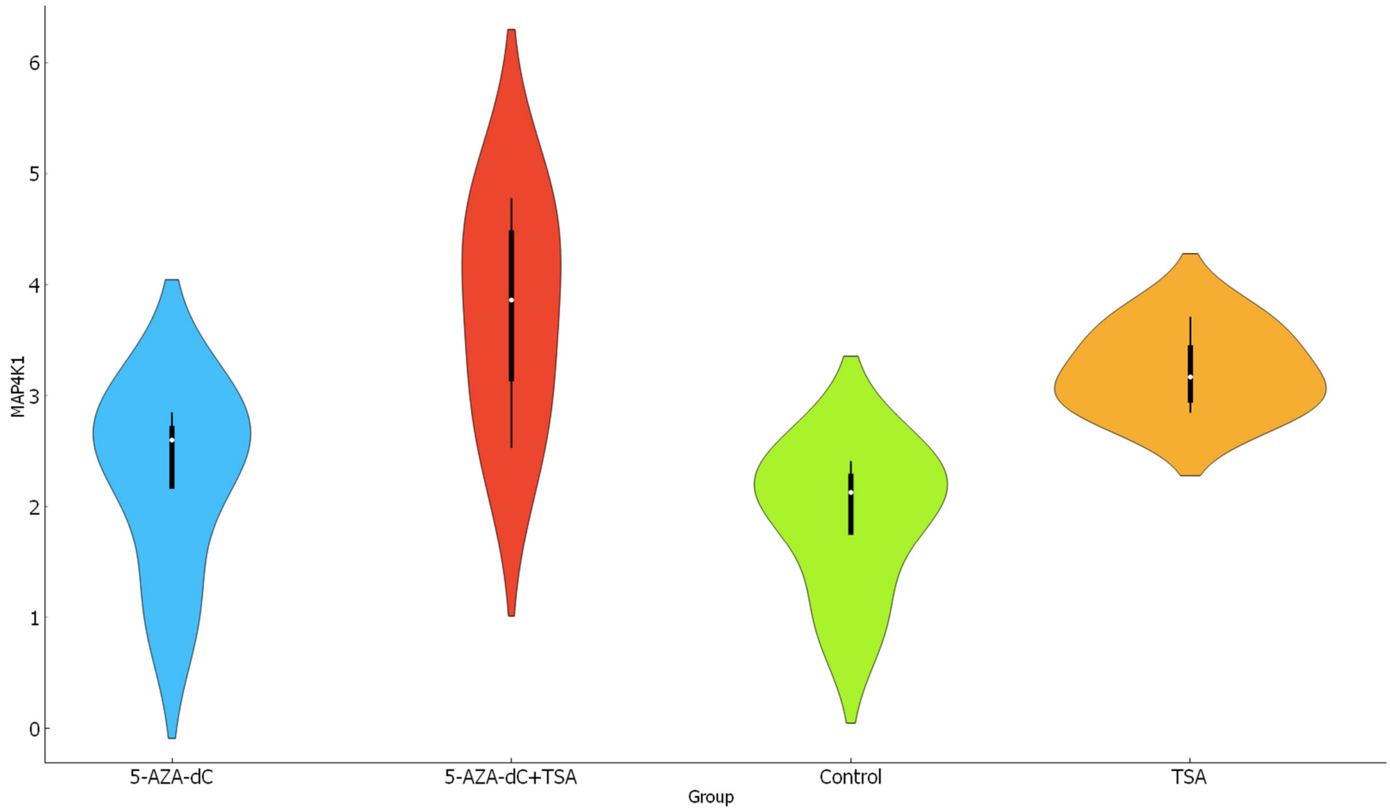

Figure S29. Results of real-time PCR analysis of MAP4K1 gene in chondrocytes dependently on applied stimulation: 5-AZA-dc, 5-AZA-dc+TSA, control and TSA. *p-value < 0.05, ** p-value < 0.01, ns - p > 0.05

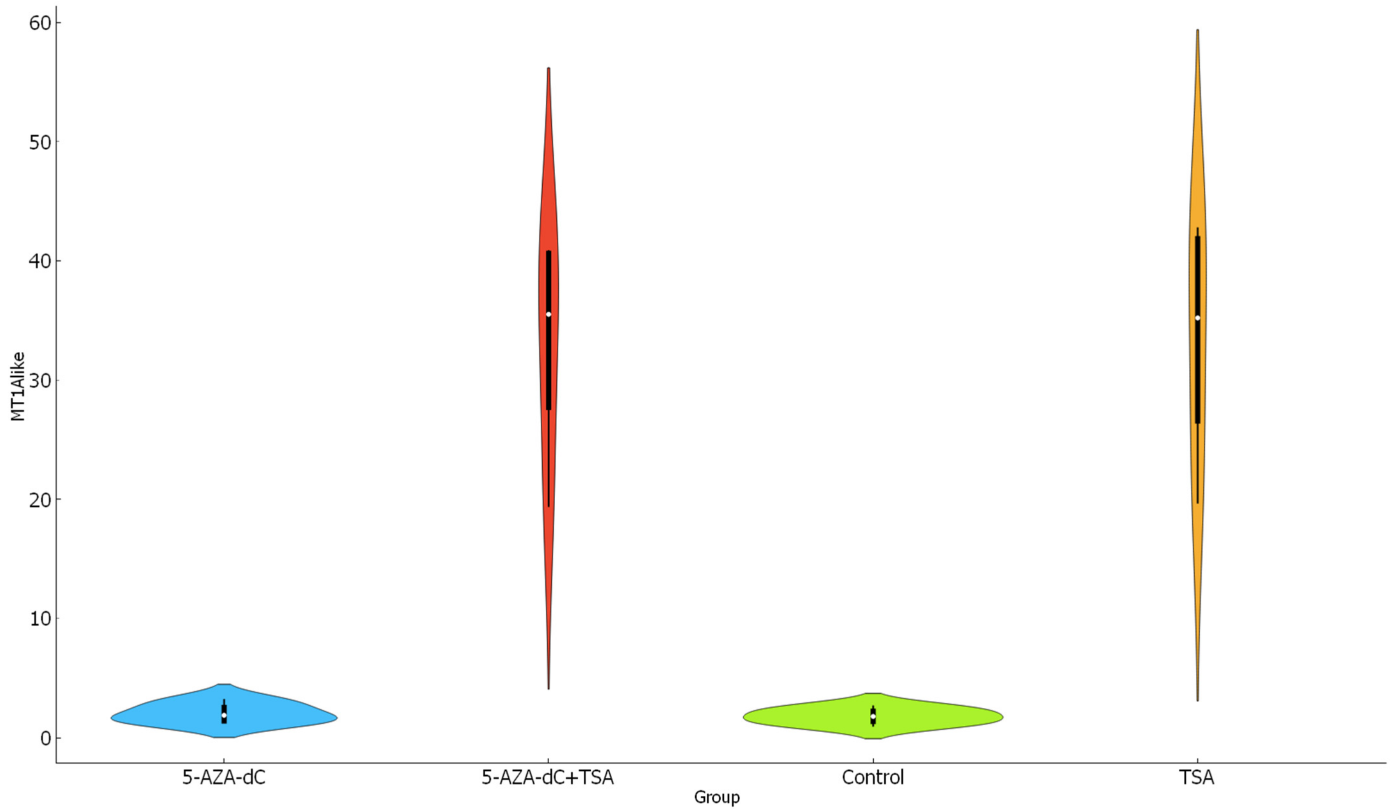

Figure S30. Results of real-time PCR analysis of MTA1 gene in chondrocytes dependently on applied stimulation: 5-AZA-dc, 5-AZA-dc+TSA, control and TSA. *p-value < 0.05, ** p-value < 0.01, ns - p > 0.05

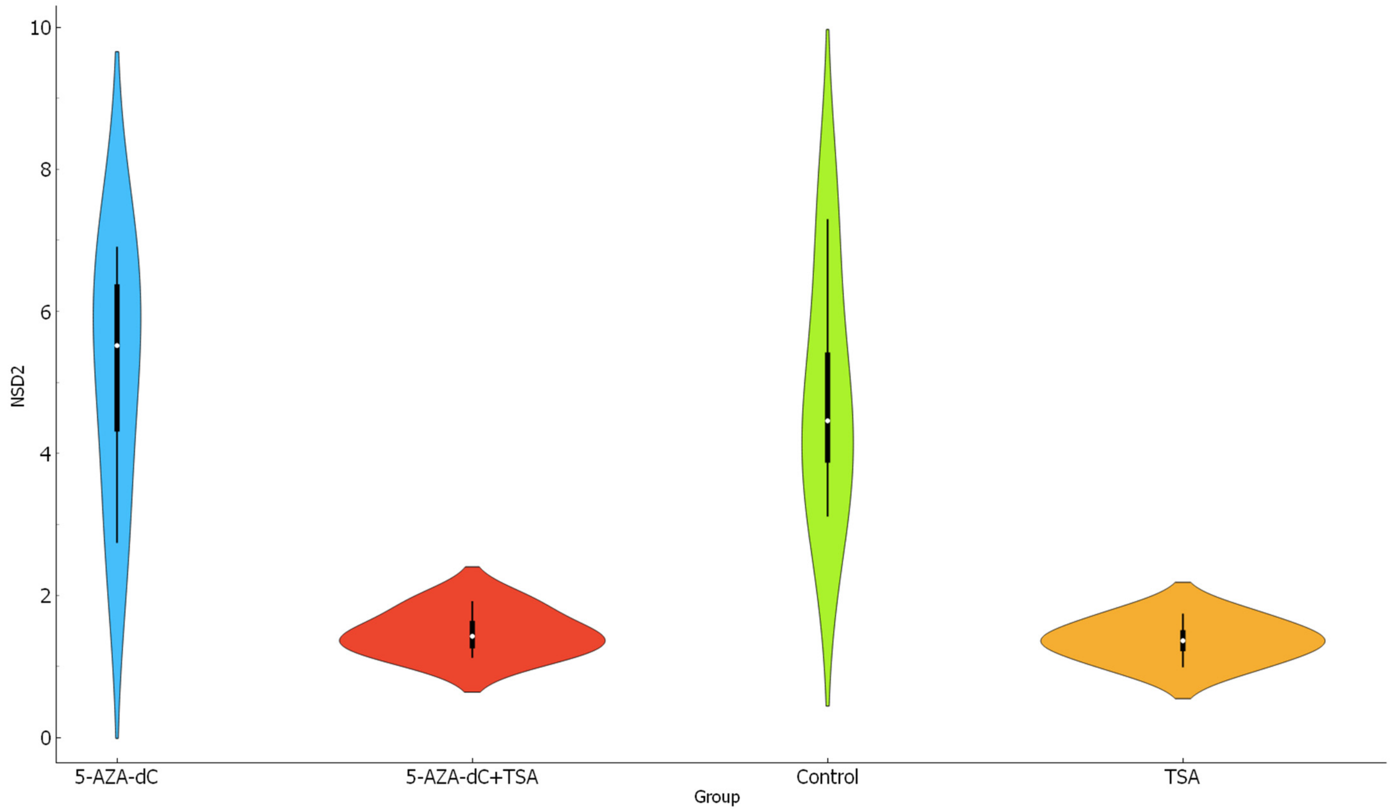

Figure S31. Results of real-time PCR analysis of NSD2 gene in chondrocytes dependently on applied stimulation: 5-AZA-dc, 5-AZA-dc+TSA, control and TSA. *p-value < 0.05, ** p-value < 0.01, ns - p > 0.05

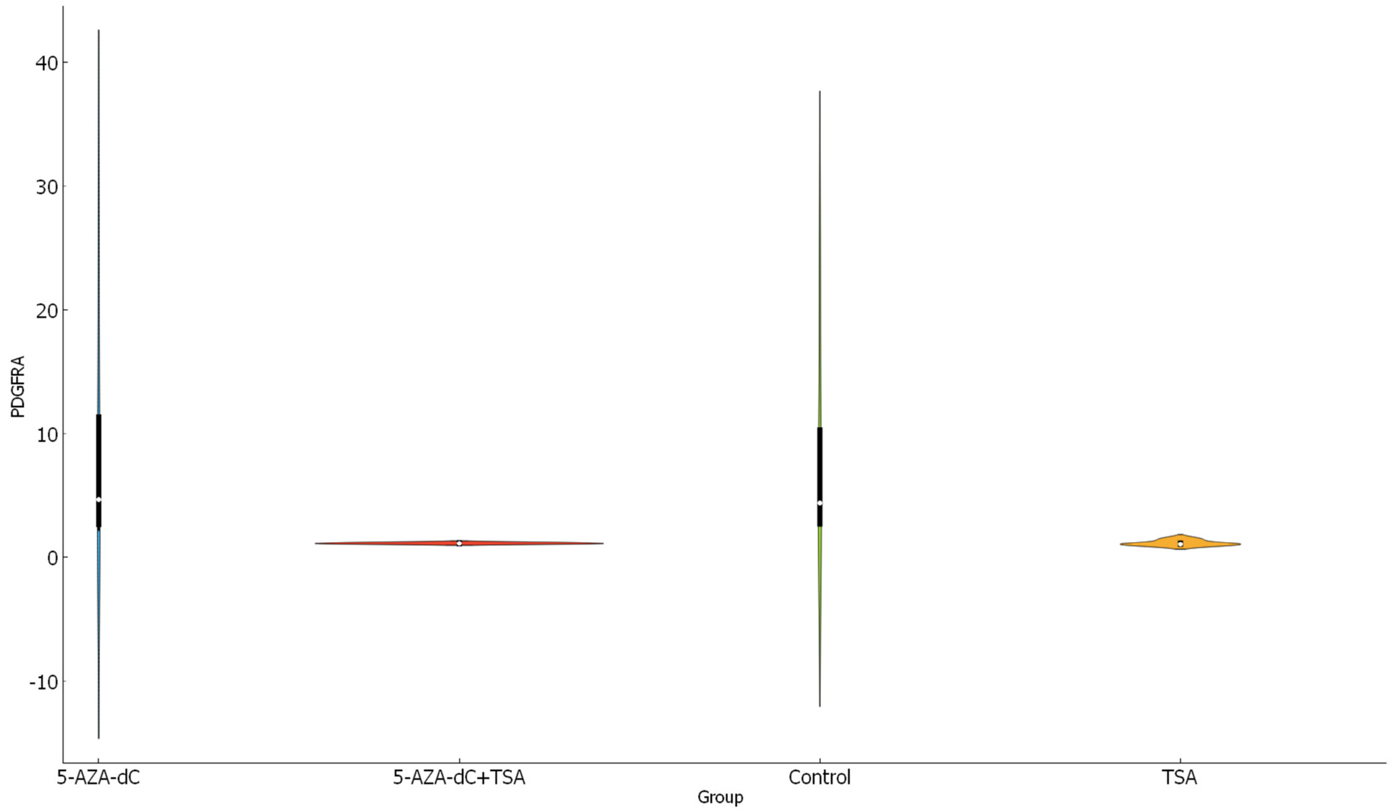

Figure S32. Results of real-time PCR analysis of PDGFRA gene in chondrocytes dependently on applied stimulation: 5-AZA-dc, 5-AZA-dc+TSA, control and TSA. *p-value < 0.05, ** p-value < 0.01, ns - p > 0.05

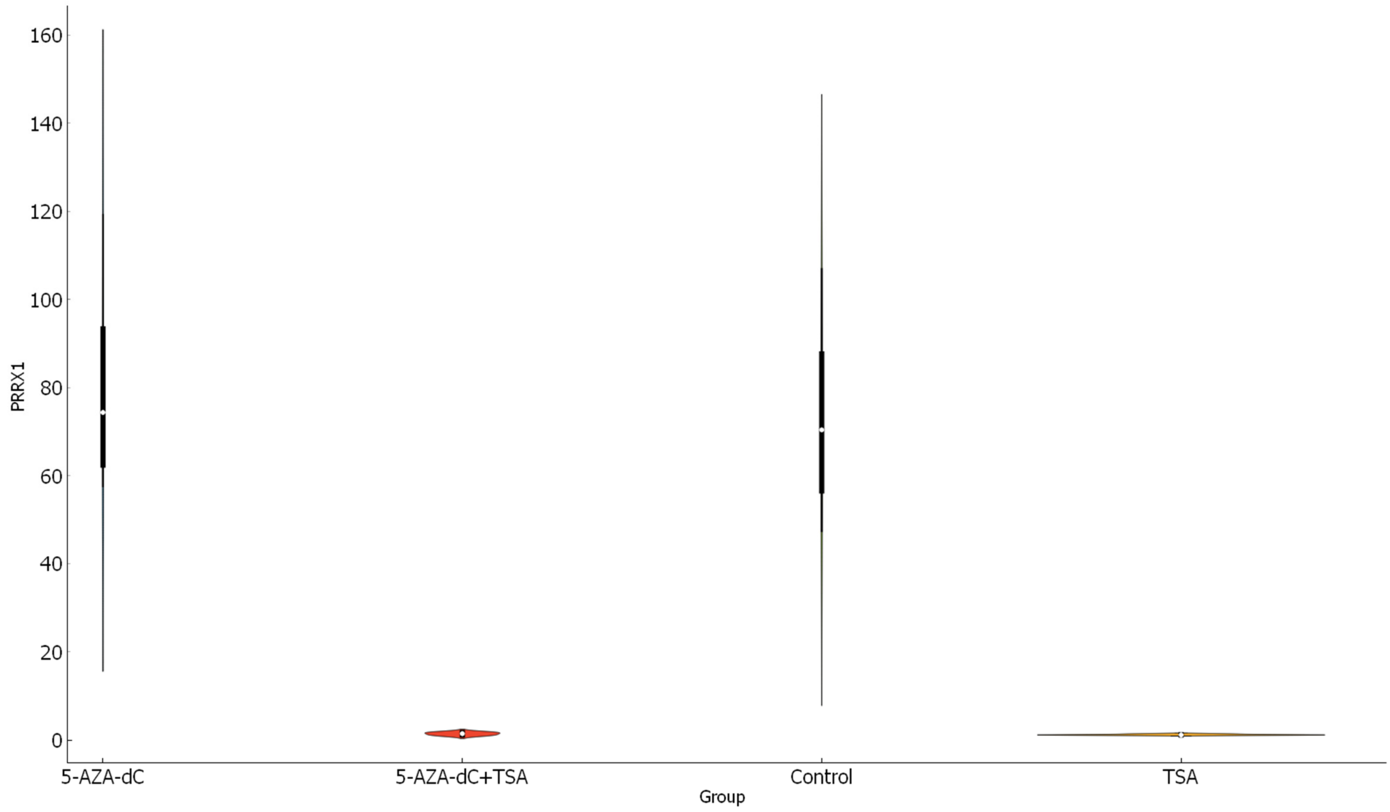

Figure S33. Results of real-time PCR analysis of PRRX1 gene in chondrocytes dependently on applied stimulation: 5-AZA-dc, 5-AZA-dc+TSA, control and TSA. *p-value < 0.05, ** p-value < 0.01, ns - p > 0.05

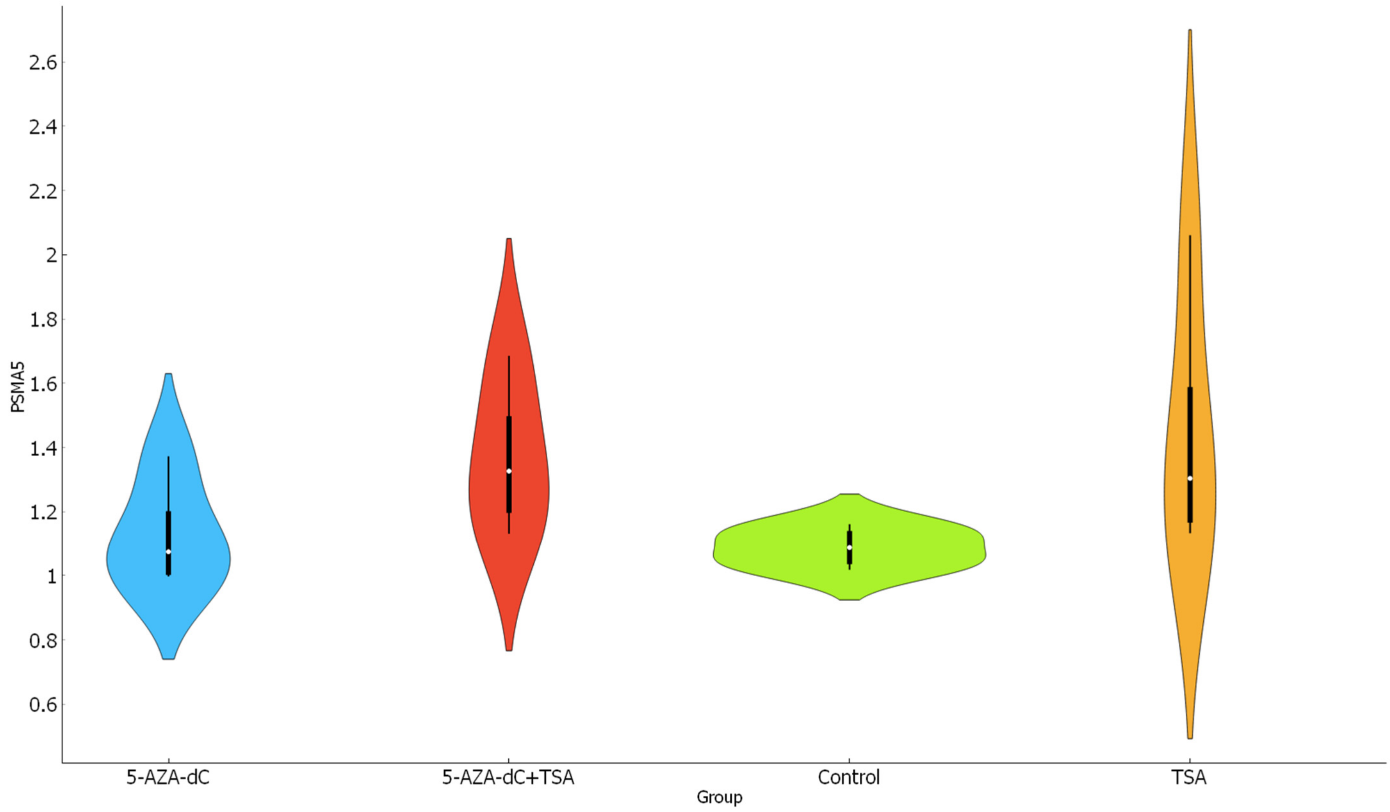

Figure S34. Results of real-time PCR analysis of PSMA5 gene in chondrocytes dependently on applied stimulation: 5-AZA-dc, 5-AZA-dc+TSA, control and TSA. *p-value < 0.05, ** p-value < 0.01, ns - p > 0.05

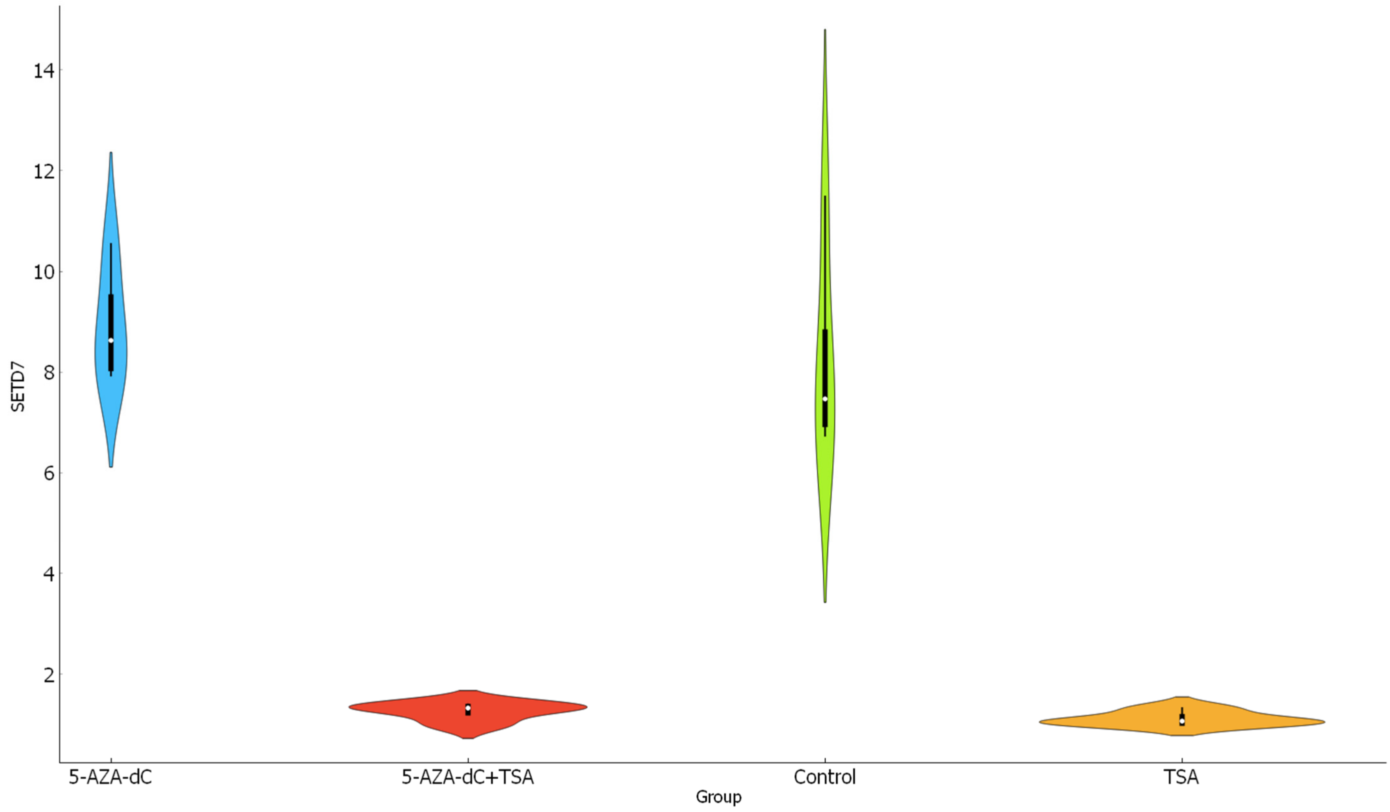

Figure S35. Results of real-time PCR analysis of SETD7 gene in chondrocytes dependently on applied stimulation: 5-AZA-dc, 5-AZA-dc+TSA, control and TSA. *p-value < 0.05, ** p-value < 0.01, ns - p > 0.05

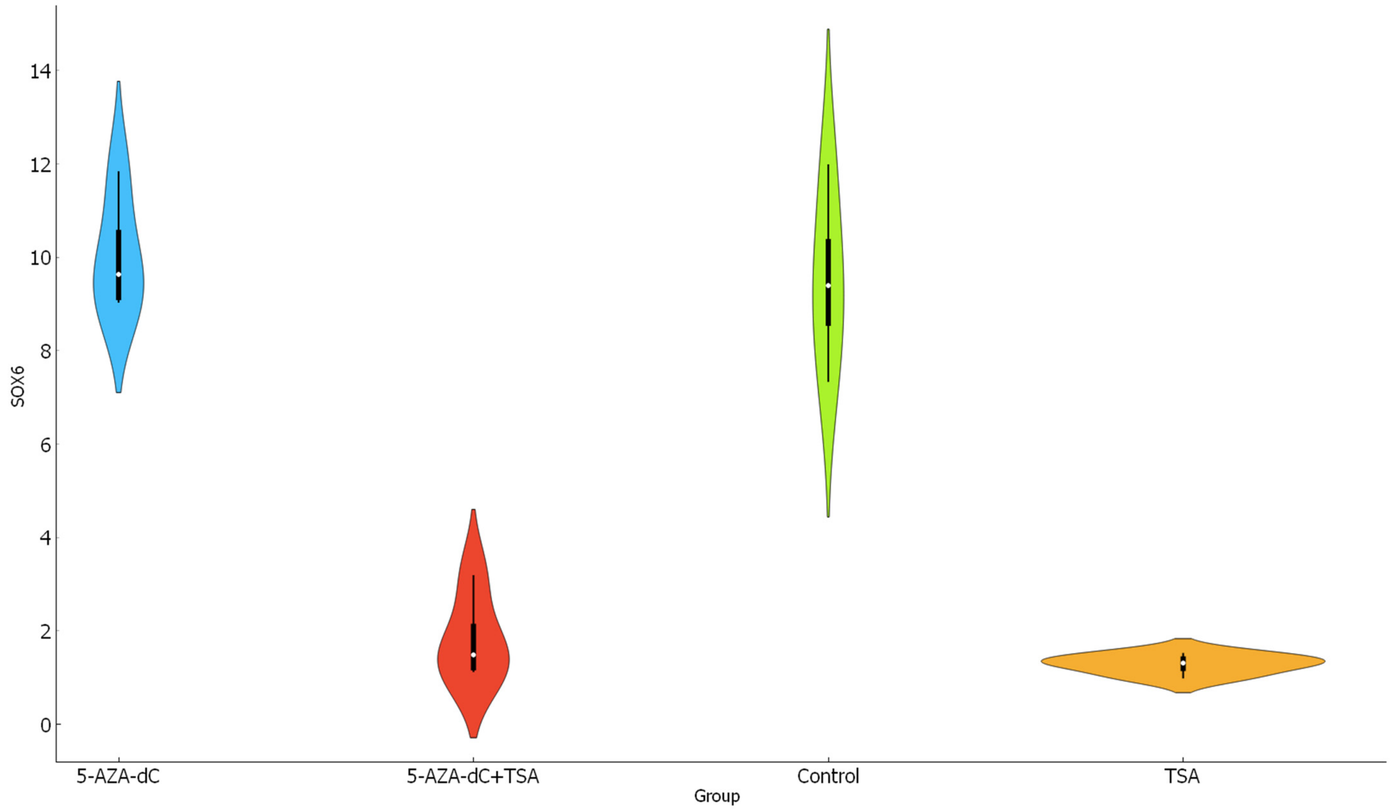

Figure S36. Results of real-time PCR analysis of SOX6 gene in chondrocytes dependently on applied stimulation: 5-AZA-dc, 5-AZA-dc+TSA, control and TSA. *p-value < 0.05, ** p-value < 0.01, ns - p > 0.05

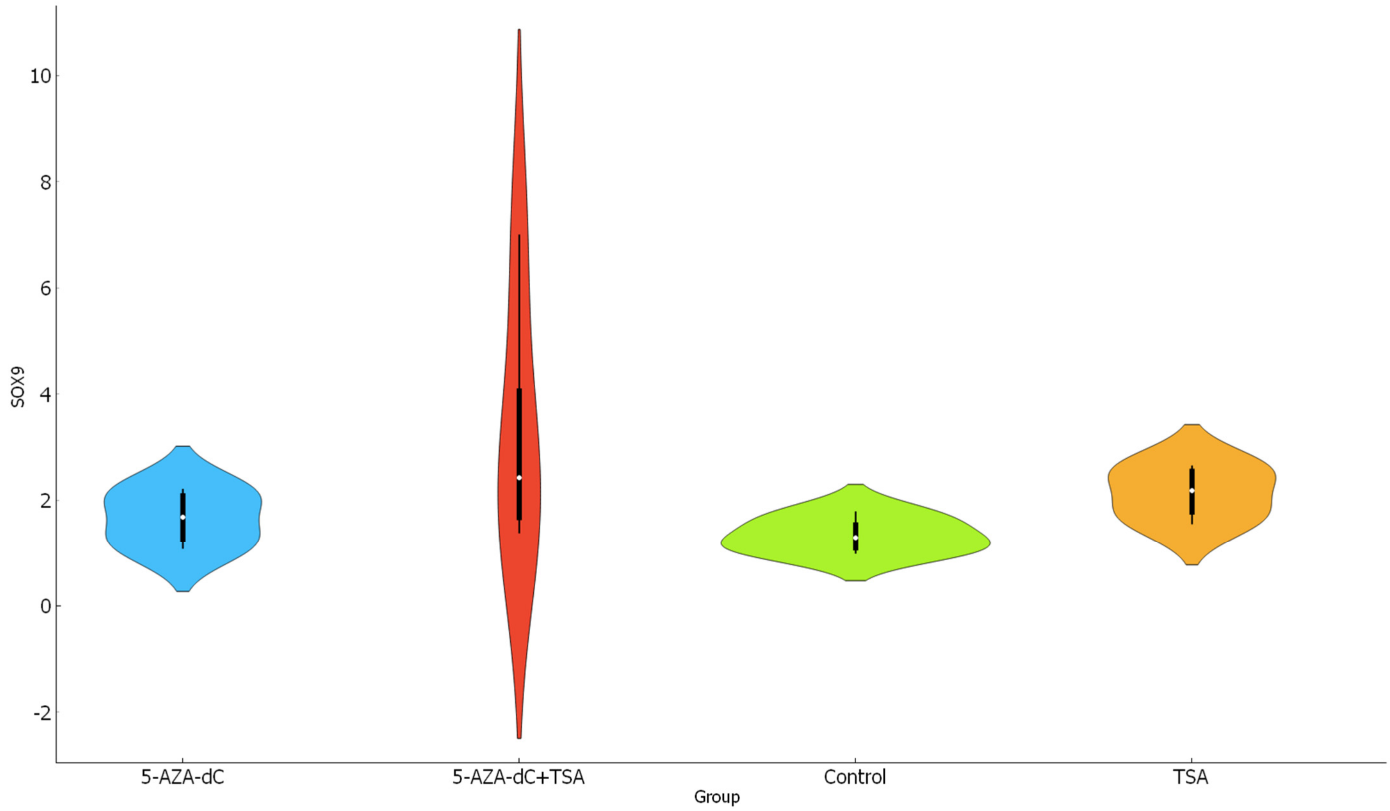

Figure S37. Results of real-time PCR analysis of SOX9 gene in chondrocytes dependently on applied stimulation: 5-AZA-dc, 5-AZA-dc+TSA, control and TSA. *p-value < 0.05, ** p-value < 0.01, ns - p > 0.05

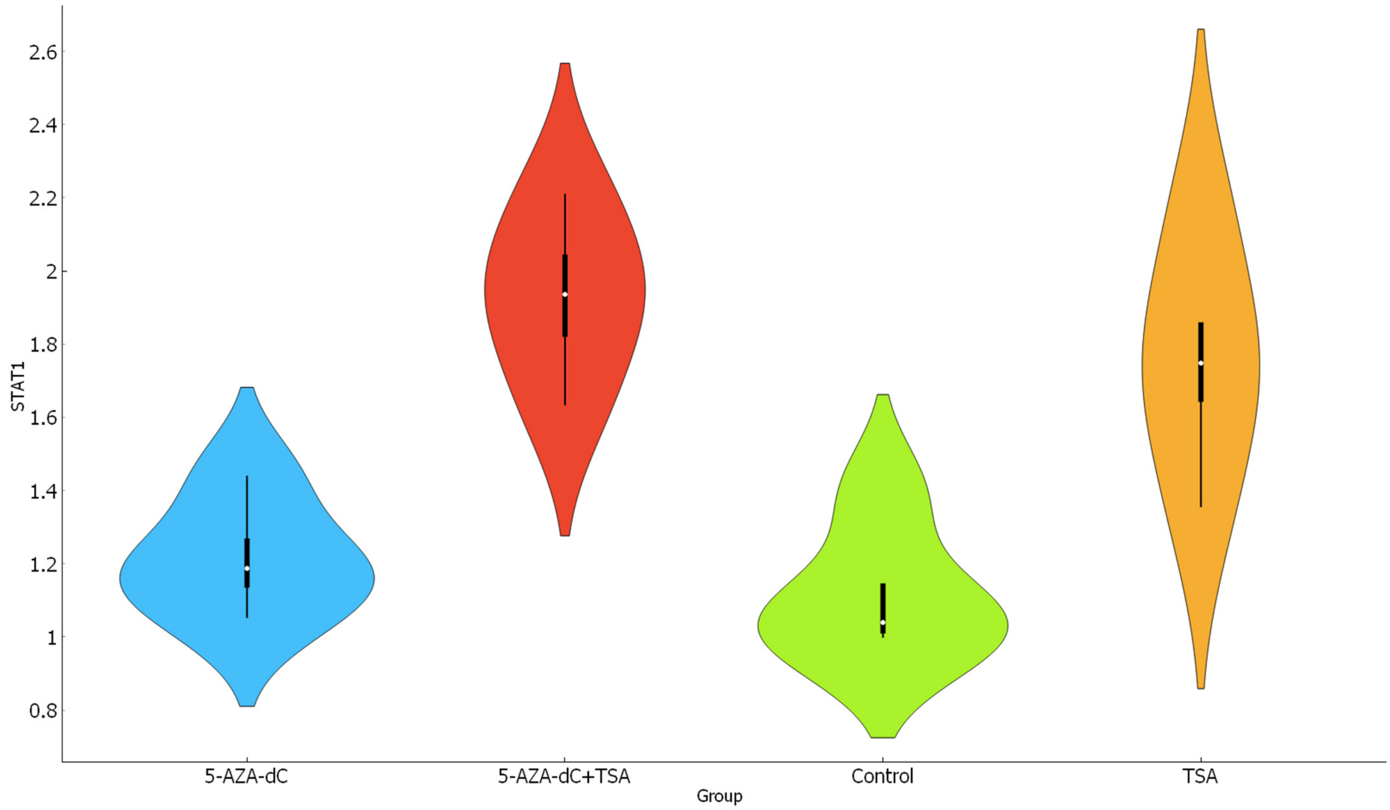

Figure S38. Results of real-time PCR analysis of STAT1 gene in chondrocytes dependently on applied stimulation: 5-AZA-dc, 5-AZA-dc+TSA, control and TSA. *p-value < 0.05, ** p-value < 0.01, ns - p > 0.05

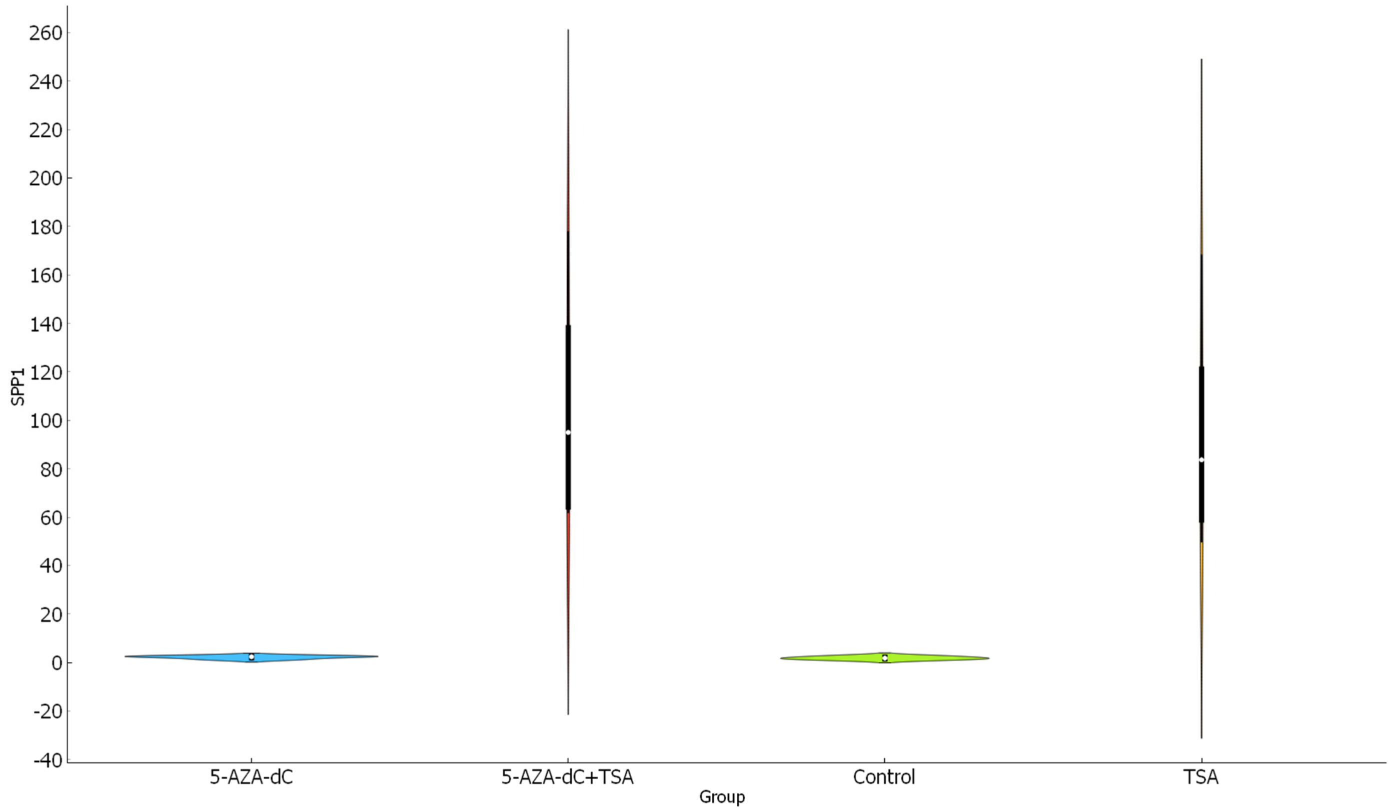

Figure S39. Results of real-time PCR analysis of SPP1 gene in chondrocytes dependently on applied stimulation: 5-AZA-dc, 5-AZA-dc+TSA, control and TSA. *p-value < 0.05, ** p-value < 0.01, ns - p > 0.05

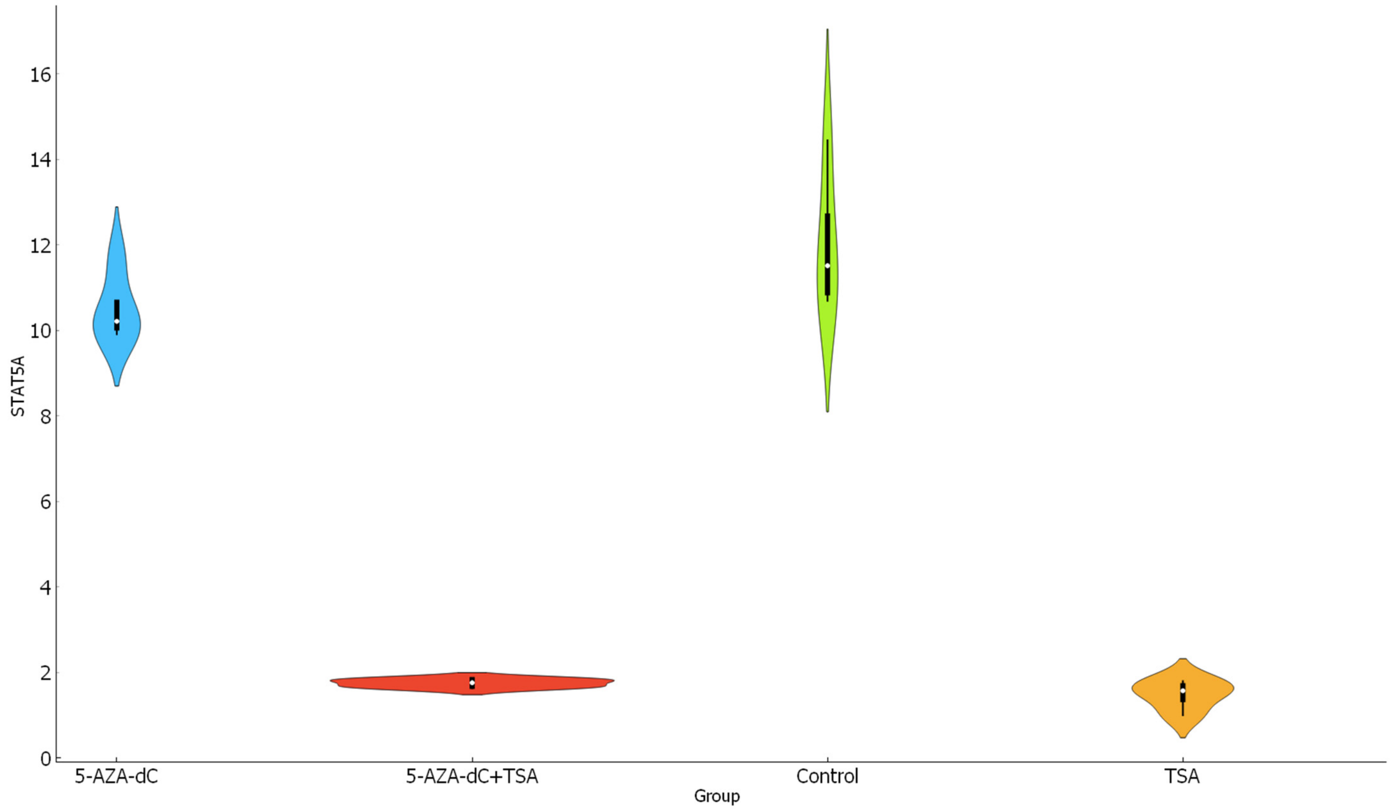

Figure S40. Results of real-time PCR analysis of STAT5A gene in chondrocytes dependently on applied stimulation: 5-AZA-dc, 5-AZA-dc+TSA, control and TSA. *p-value < 0.05, ** p-value < 0.01, ns - p > 0.05

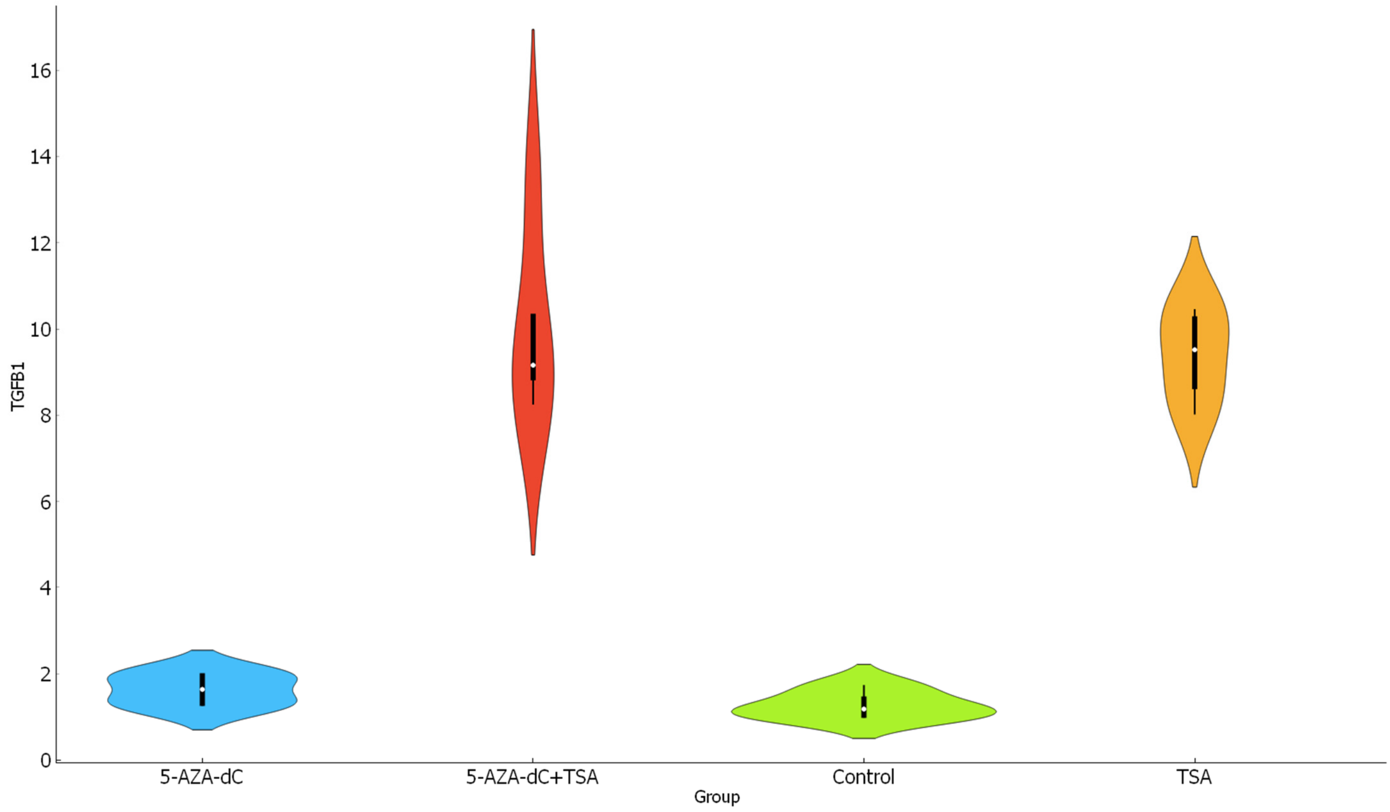

Figure S41. Results of real-time PCR analysis of TGFB1 gene in chondrocytes dependently on applied stimulation: 5-AZA-dc, 5-AZA-dc+TSA, control and TSA. *p-value < 0.05, ** p-value < 0.01, ns - p > 0.05

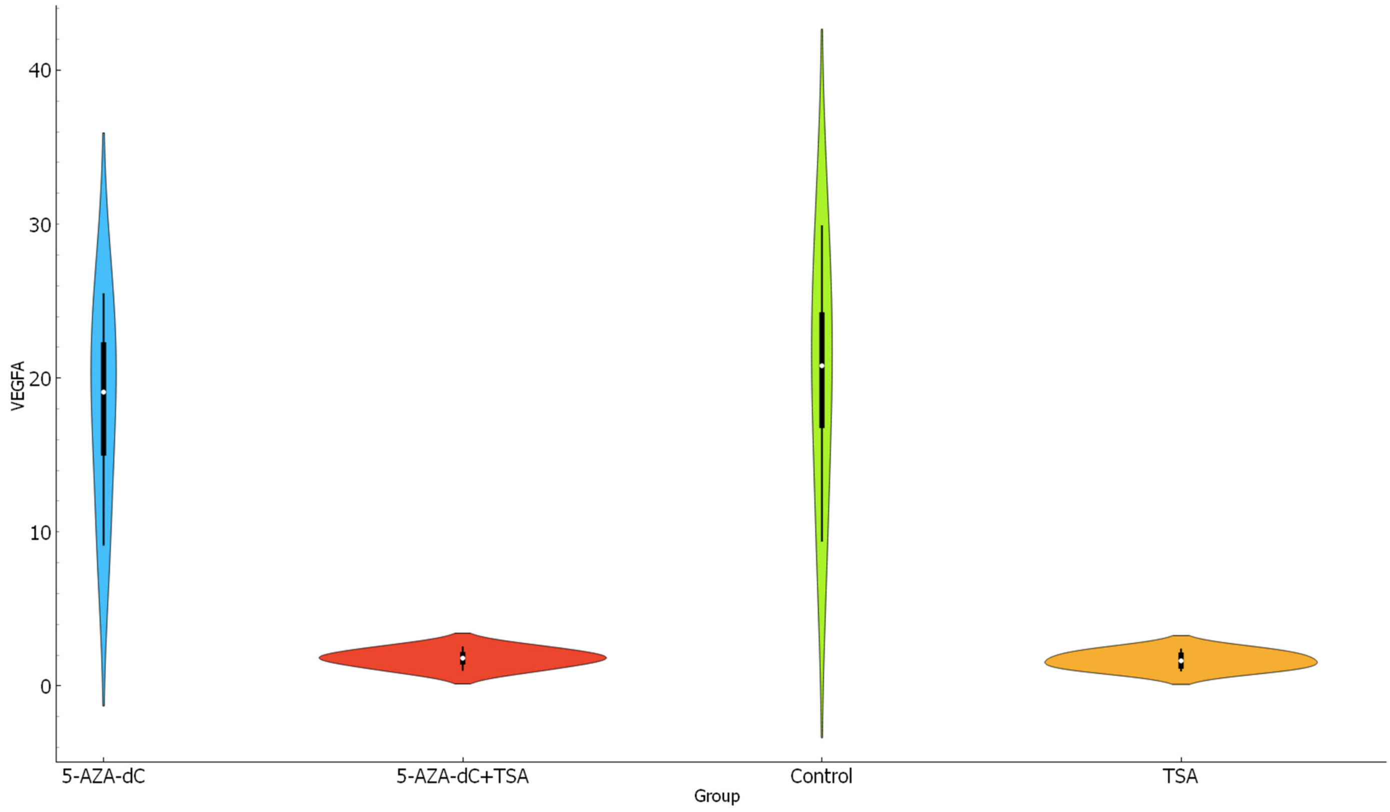

Figure S42. Results of real-time PCR analysis of VEGFA gene in chondrocytes dependently on applied stimulation: 5-AZA-dc, 5-AZA-dc+TSA, control and TSA. *p-value < 0.05, ** p-value < 0.01, ns - p > 0.05
